# Identification, characterization and expression profiles of *Fusarium udum* stress-responsive *WRKY* transcription factors in *Cajanus cajan* under the influence of NaCl stress and *Pseudomonas fluorescens* OKC

**DOI:** 10.1038/s41598-019-50696-x

**Published:** 2019-10-04

**Authors:** Gagan Kumar, Raina Bajpai, Ankita Sarkar, Raj Kumar Mishra, Vijai Kumar Gupta, Harikesh B. Singh, Birinchi K. Sarma

**Affiliations:** 1 Department of Mycology and Plant Pathology, Institute of Agricultural Sciences, Banaras Hindu University, Varanasi, 221005 India; 2 ICAR-Indian Institute of Pulses Research, Kanpur, 208024 India; 3 Department of Chemistry and Biotechnology, Tallinn University of Technology, Tallinn, Estonia

**Keywords:** Gene expression profiling, Gene expression

## Abstract

The WRKY gene family has never been identified in pigeonpea (*Cajanus cajan*). Therefore, objective of the present study was to identify the WRKY gene family in pigeonpea and characterize the *Fusarium udum* stress-responsive WRKY genes under normal, NaCl-stressed and *Pseudomonas fluorescens* OKC (a plant growth-promoting bacterial strain) treated conditions. The aim was to characterize the *Fusarium udum* stress*-*responsive WRKY genes under some commonly occurring field conditions. We identified 97 genes in the WRKY family of pigeonpea, using computational prediction method. The gene family was then classified into three groups through phylogenetic analysis of the homologous genes from the representative plant species. Among the 97 identified WRKY genes 35 were further classified as pathogen stress responsive genes. Functional validation of the 35 WRKY genes was done through generating transcriptional profiles of the genes from root tissues of pigeonpea plants under the influence of *P*. *fluorescens* OKC after 24 h of stress application (biotic: *Fusarium udum*, abiotic: NaCl). The entire experiment was conducted in two pigeonpea cultivars Asha (resistant to *F*. *udum*) and Bahar (susceptible to *F*. *udum*) and the results were concluded on the basis of transcriptional regulation of the WRKY genes in both the pigeonpea cultivars. The results revealed that among the 35 tentatively identified biotic stress responsive CcWRKY genes, 26 were highly *F*. *udum* responsive, 17 were better NaCl responsive compared to *F*. *udum* and 11 were dual responsive to both *F*. *udum* and NaCl. Application of OKC was able to enhance transcript accumulation of the individual CcWRKY genes to both the stresses when applied individually but not in combined challenge of the two stresses. The results thus indicated that CcWRKY genes play a vital role in the defense signaling against *F*. *udum* and some of the *F*. *udum* responsive CcWRKYs (at least 11 in pigeonpea) are also responsive to abiotic stresses such as NaCl. Further, plant beneficial microbes such as *P*. *fluorescens* OKC also help pegionpea to defend itself against the two stresses (*F*. *udum* and NaCl) through enhanced expression of the stress responsive CcWRKY genes when the stresses are applied individually.

## Introduction

The WRKY family of transcription factors (TFs) is one of the most important gene families in higher plants involved in biotic and abiotic stress responses^1^. As TFs, WRKY gene family acts with other components of the transcriptional machinery and plays a vital role in the plant response to both external and internal stimuli^2,3^. Identification and characterization of WRKY genes in *Jatropha curcas* revealed presence of 47 WRKY genes that showed responsiveness to one or more abiotic stresses^4^. Similarly, the Arabidopsis WRKY gene *AtWRKY25* is induced in response to the bacterial pathogen *Pseudomonas syringae* and plays a role in the host defense^5^. Other Arabidopsis WRKY genes such as, *AtWRKY3*, *AtWRKY4* and *AtWRKY8* demonstrated to respond to the necrotrophic fungal pathogen *Botrytis cinerea*^6,7^. It is also well documented that a single gene may play a specific role at a varied level against a specific stress under different circumstances. WRKY proteins possess one or two unique DNA binding domains that consist of about 60 amino acid long peptide sequences including the highly conserved sequence WRKYGQK and a zinc-finger motif. WRKY family proteins have been classified into three groups (I-III) based on the WRKY domain numbers as well as structure of the zinc-finger motif. WRKY proteins in group I possess two WRKY domains along with a C_2_H_2_ like zinc-finger motif. Group II WRKY proteins possess a single WRKY domain along with a C_2_H_2_ like zinc-finger motif. Based on the type of conserved motifs group II can be further sub-divided into five different groups (IIa–IIe)^8^. Finally, group III WRKY proteins possess a single WRKY domain in addition to a C_2_HC type zinc-finger motif. Evolutionary studies indicated that the majority of WRKY gene members in groups I and II were emerged before the divergence of monocot and dicot plants, whereas group III genes appeared relatively later^9^.

WRKY genes have been identified and characterized in various plant species^8–11^, since the first cDNA of the WRKY gene *SPF1* was cloned from sweet potato^12^. Different plant species carry different numbers of WRKY genes. For example, 72 WRKY family members have been identified in the Arabidopsis genome^9^, 102 in rice^9^, 55 in cucumber^13^, 119 in maize^14^, 59 in grapevine^15^, 104 in poplar^16^, etc. In addition to higher plants, WRKY-type genes were also reported in non-plant green alga and other eukaryotes^17^.

Pigeonpea (*Cajanus cajan* (L.) Millsp.) is a widely cultivated food legume in South Asia, Africa, the Caribbean and Latin America, where it is considered as an important source of protein in the human diet^18^. *Fusarium udum* is a very serious wilt pathogen for pigeonpea and the role of the pigeonpea WRKY genes against *F*. *udum* or any other pathogen stress is not yet investigated. Therefore, the aim of the present study is to (i) identify and validate the tentative WRKY TFs of pigeonpea that respond to *F*. *udum* challenge, (ii) considering the increasing areas of salinity in cultivable lands, the same set of WRKY genes was also evaluated for their responsiveness to NaCl stress either individually or in combination with *F*. *udum* and (iii) a plant growth-promoting rhizobacteria (PGPR) *Pseudomonas fluorescens* OKC, a potential plant stress reliever, was used to see its impact on the selected *WRKY* genes under both *F*. *udum* and NaCl stresses in pigeonpea.

## Results

### Identification and phylogenetic analysis of pigeonpea WRKY genes

**Figure 1:**
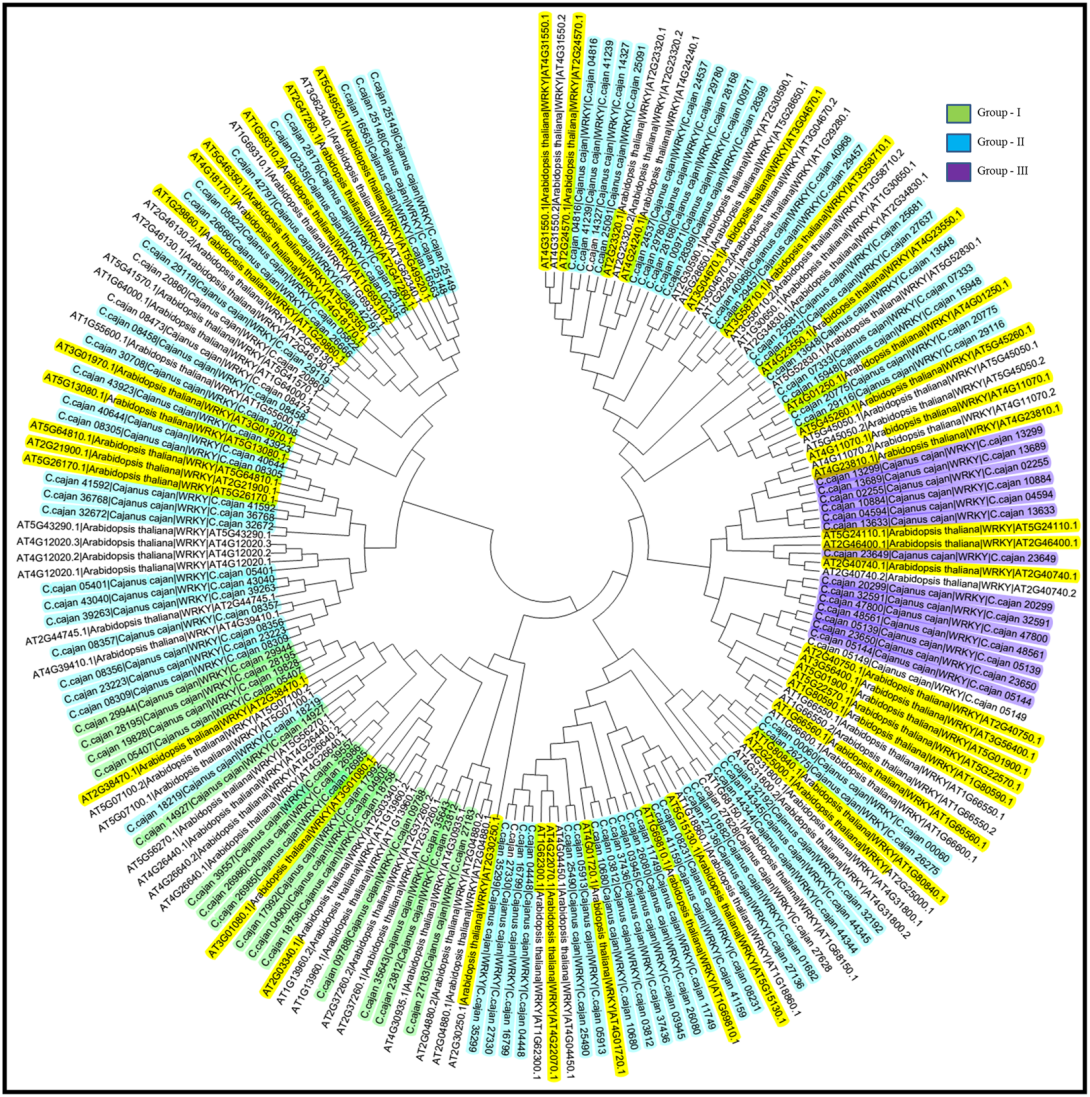
Phylogenetic tree of the WRKY proteins of pigeonpea (*Cajanus cajan*). The conserved WRKY domain sequences of pigeonpea WRKY proteins were used to construct multiple sequence alignments using Clustal W. Software MEGA was used to prepare the phylogenetic tree and neighbor-joining method was adopted by 1,000 bootstrap replications. The CcWRKY proteins of pigeonpea are classified into three groups, Group I–III, based on conserved WRKY domain sequences. Yellow colour indicates the *Arabidopsis* WRKY defense responsive genes used for selection of pigeonpea WRKY defense responsive genes.

Sequence analysis of the conserved domains in pigeonpea led to identification of 97 WRKY genes (further defined as CcWRKY genes) (Supplementary Table S1). All putative 97 CcWRKY proteins in pigeonpea and 72 WRKY proteins in *Arabidopsis thaliana* were used to perform phylogenetic study to categorize and investigate the evolutionary relationships of the CcWRKY genes. Phylogenetic relationship study showed that the CcWRKY proteins in pigeonpea could be categorized into three groups, Group I–III, which are highly conserved in both monocots and eudicots^8^ (Fig. 1). A total of 11 CcWRKY proteins are placed in group I with two WRKY domains and C_2_H_2_-type of zinc-finger motif, 69 CcWRKY proteins that contained one WRKY domain and also C_2_H_2_-type zinc binding motif is placed in group II and the remaining 14 CcWRKY proteins with a single WRKY domain and C_2_HC type of zinc-binding motif is assigned to group III. Out of the 97 CcWRKY genes, 3 sequences (C.cajan_08356, C.cajan_08357, C.cajan_47800) were devoid of any WRKY domain and zinc binding motif. These exceptional WRKY proteins could represent pseudogenes or sequencing and/or assembly errors^19,20^.

### *In silico* functional analysis of CcWRKY proteins

We retrieved a total of 97 protein sequences as members of the WRKY family in *C*. *cajan*. A homology search was carried out using BLAST in NCBI and TAIR databases, and all CcWRKY proteins showed sequence identity with WRKY proteins from *Arabidopsis thaliana*. *In silico* functional analysis showed that 56 CcWRKY proteins are similar to either biotic or abiotic stress-related AtWRKY proteins in *A*. *thaliana* and 35 of them are pathogen stress responsive (Supplementary Table S2). Thus, we assumed that a great number of the CcWRKY proteins are also involved in response to various biotic and abiotic stresses.

### Motif analysis of CcWRKY Genes

In the CcWRKY family a total of 25 motifs were identified in different CcWRKY proteins (Supplementary Fig. SF1, Supplementary Table S3). Results from the MEME motif analysis showed that the CcWRKY proteins have variation in occurrence of motifs numbers (1–9), and also in the length of motifs (ranged from 7 to 95 amino acids long). However, 3 motifs out of the 25 namely motifs 1, 2 and 8 showed presence of the most conserved sequence WRKYGQK. The 22 remaining motifs were located outside the WRKY domain. Further, motif 1 is shared by 45 CcWRKYs belonging to all three groups while motif 2 is shared by 47 CcWRKYs belonging to group I and group II only and motif 8 is shared by 3 CcWRKY genes from group II (*CcWRKY35*, *CcWRKY5*7, *CcWRKY73*) and 6 CcWRKY genes from group III (*CcWRKY7*, *CcWRKY*34, *CcWRKY49*, *CcWRKY66*, *CcWRKY86*, *CcWRKY97*). In addition, motifs 1, 3 and 4 are those which are shared by the members of all three groups while motifs 2, 5, 9 and 14 are shared by the members of group I and II only. Members of the group II and III are shared by motifs 8, 13, 15, 19 and 24 whereas, motifs 6, 7, 10, 12, 16, 20, 22 and 23 are found conserved only in the group II CcWRKY members and motifs 21 and 25 are conserved in the members of group III only. The motifs 1 and 2 showed similarity with *AtWRKY4* which is involved in defense responses such as negative regulation of defense response to bacterium, regulation of defense response to fungus, regulation of transcription, DNA-templated, response to ethylene, jasmonic acid and salicylic acid^21^ while motif 8 showed similarity with *AtWRKY1* which is linked to positive regulation of transcription, DNA-templated, and salicylic acid mediated signaling pathway^22^. All the 35 pathogen stress responsive CcWRKYs had one of the above mentioned motifs and therefore confirmed to be CcWRKYs. In the selected CcWRKYs, motif 24 (RGRHTCT) is found only in *CcWRKY*s *7*, *34* and 35 that showed similarity with TOPLESS (TPL) and TOPLESS-related (TPR) proteins. In plants, TPL/TPR are co-repressors that regulate development, stress responses, and hormone signaling through interaction with small ethylene response factor-associated amphiphilic repression (EAR) motifs and normally found in diverse transcriptional repressors^23^ (Supplementary Table S3).

### Protein structure analysis of CcWRKY

The protein structures of CcWRKYs were further analyzed and the results showed that 84 CcWRKYs contained the highly conserved sequence WRKYGQK whereas proteins of 5 CcWRKYs (CcWRKY21, CcWRKY22, CcWRKY23, CcWRKY24 and CcWRKY57) consist of the most common variant sequence WRKYGKK. Proteins of four CcWRKYs (CcWRKY19, CcWRKY34, CcWRKY35 and CcWRKY84) consist of the less common variant sequence WRKYGEK. CcWRKY50 protein consists of four and CcWRKY13 consists of one amino acid variation in their WRKY domains. In three CcWRKY genes the WRKY domain was not found (Supplementary Fig. SF2).

### Physico-chemical characterization of CcWRKY proteins

Amino acid residues calculated from the primary sequences of the CcWRKY proteins ranged from 66 to 678 (Supplementary Table S4). Isoelectric points of all CcWRKYs ranged between 4.74 and 10.09 and fifty percent of the proteins are acidic in nature (pI < 7) and fifty percent showed alkaline nature (pI > 7). Molecular weight of the proteins was between 7935.92 and 73688.00 Da. Almost all proteins except seven have instability index greater than 40, which indicated that the proteins are unstable in nature. The aliphatic index of all the proteins is significantly high which may be regarded as a positive factor for increased thermostability of globular proteins^24^. Lower range of GRAVY indicated that the proteins have better interaction with water.

### Transcript profiling of CcWRKY genes against biotic and abiotic stresses

**Figure 2:**
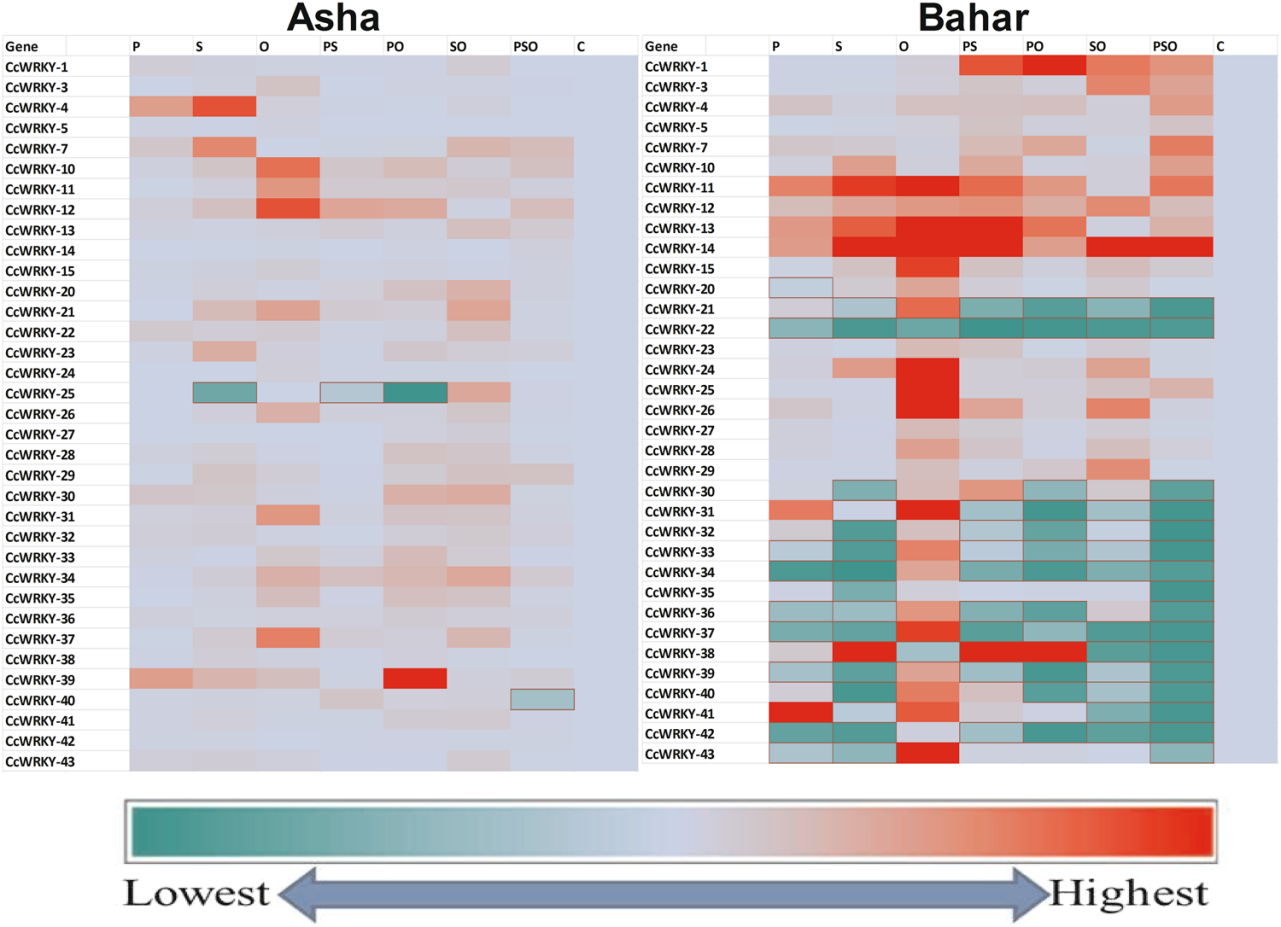
Heat maps of expression profiles of 35 selected CcWRKY genes in the pigeonpea cultivars Asha and Bahar in different treatments: P (Pathogen *Fusarium udum*), S (Salt-NaCl), O (*Pseudomonas fluorescens* OKC), PS (Pathogen + Salt), PO (Pathogen + *Pseudomonas*), SO (Salt + *Pseudomonas*), PSO (Pathogen + Salt + *Pseudomonas*) and C (Control). The colour scale indicates the expression value (dark red indicate higher expression value, darker green indicates lower gene expression values).

*In silico* functional analysis of the 97 pigeonpea WRKY genes showed that 35 are pathogen stress responsive genes. Quantification analyses of the CcWRKY gene transcripts in response to the biotic (*Fusarium udum* WSP-V2) and abiotic (NaCl) challenges revealed differential expression patterns of the 35 CcWRKY genes (Fig. 2, Supplementary Tables S5 and S6). Details of expression profiles of the genes in the two pigeonpea cultivars are described with the internal control Actin as both the internal controls (Actin and β-tubulin) provided similar trends of expression profiles of the selected genes and expression profiles of the individual genes were statistically not different between the two internal controls.

### Transcript profiling of the CcWRKY genes in response to *Fusarium udum* in resistant and susceptible cultivars

Transcript profiling of the WRKY genes was done in both the *F*. *udum* resistant and susceptible pigeonpea cultivars ‘Asha’ and ‘Bahar’, respectively, to observe the cultivar-specific response to *F*. *udum* challenge. In pigeonpea, all 35 CcWRKY genes did not respond equally to *F*. *udum* stress in the two cultivars. In the resistant cultivar ‘Asha’ all 35 CcWRKY’s were up-regulated but in different folds under the pathogen stress, viz., *CcWRKY*s *3*, *5*, *11*, *15*, *20*, *21*, *23*, *24*, *25*, *26*, *27*, *29*, *34*, *35*, *37*, *38*, *40*, and 42 were up-regulated by 1–5 fold, whereas *CcWRKY*s 10, 13, 28, 31, 33, 36, and 41 by 5–10 fold, *CcWRKY*s 1, 12, 32, and 43 by 10–15 fold, *CcWRKY*s7 and 22 by 15–20 fold, *CcWRKY30* by 20 fold while two *CcWRKY*s 4 and 39 by more than 50 fold. However, 9 CcWRKYs (*CcWRKY*s 3, 14, 21, 24, 25, 26, 27, 29, and 35) were least responsive to *F*. *udum*. These CcWRKYs were up-regulated minimally (0.5–1 fold). At the same time CcWRKYs in the susceptible cultivar ‘Bahar’ revealed that 9 CcWRKYs (*CcWRKY*s *20*, *22*, *33*, *34*, *36*, *37*, *39*, *42*, and 43) were down-regulated and only 26 CcWRKYs were up-regulated. Among the up-regulated ones 21 CcWRKYs (*CcWRKY*s *1*, *3*, *4*, *5*, *7*, *10*, *12*, *15*, *21*, *23*, *24*, *25*, *26*, *27*, *28*, *29*, *30*, *32*, *35*, *38*, and 40) were up-regulated by 1–5 fold, 2 (*CcWRKY*s 12, and 13) by 5–10 fold, 2 (*CcWRKY*s 11, and 31) by 10–15 fold and one (*CcWRKY41*) by 29 fold under *F*. *udum* stress (Fig. 2, Supplementary Tables S5 and S6). It was clearly observed that not all 35 selected CcWRKYs responded to *F*. *udum* in the susceptible cultivar ‘Bahar’ unlike in the case of the resistant cultivar ‘Asha’.

### Transcript profiling of the CcWRKY genes in response to NaCl stress

Transcript profiling of the WRKY genes was done in both the *F*. *udum* resistant and susceptible pigeonpea cultivars ‘Asha’ and ‘Bahar’, respectively, to observe the cultivar-specific response to NaCl challenge. Transcript profiling of all 35 pathogen responsive CcWRKYs in the two pigeonpea cultivars revealed that, in ‘Asha’ 34 genes were up-regulated viz., *CcWRKY*s *5*, *14*, *24*, *27*, *33*, and 42 by 1–5 fold, *CcWRKY*s *1*, *3*, *11*, *13*, *15*, *20*, *26*, *35*, *36*, *40*, and 41 by 5–10 fold, *CcWRKY*s *22*, *28*, *31*, *32*, *34*, *38*, and 43 by 10–15 fold, *CcWRKY*s 30, and 37 by 15–20 fold, *CcWRKY*s *10*, *12*, and 29 by 20–25 fold, *CcWRKY21* by 25–30 fold, *CcWRKY39* by 35 fold, *CcWRKY23* by 41 fold, and *CcWRKY*s 4 and 7 by more than 50 fold. Only one gene (*CcWRKY25*) was down-regulated. In contrast, 15 CcWRKY genes viz., *CcWRKY*s *21*, *22*, *30*, *31*, *32*, *33*, *34*, *35*, *36*, *37*, *39*, *40*, *41*, *42*, and 43 were down-regulated in ‘Bahar’ whereas the rest of the 20 CcWRKY genes were up-regulated. Very few CcWRKY genes viz., *CcWRKY*s *11*, *13*, *14*, *24*, *25*, and 38 in ‘Bahar’ showed higher transcript accumulation than ‘Asha’ whereas rest of the genes in ‘Bahar’ showed lower transcript accumulation compared to ‘Asha’. Interestingly, all selected 35 CcWRKYs are presumed to be *F*. *udum* responsive, but 17 of those CcWRKYs (*CcWRKY*s *7*, *10*, *11*, *12*, *15*, *20*, *21*, *23*, *26*, *28*, *29*, *31*, *34*, *35*, *37*, *38* and 40) were observed to be better responsive to NaCl compared to *F*. *udum* in the resistant cultivar ‘Asha’. (Fig. 2, Supplementary Tables S5 and S6).

### Transcript profiling of the CcWRKY genes in *Pseudomonas fluorescens* OKC treatment

Transcript profiling of the WRKY genes was done in both the *F*. *udum* resistant and susceptible pigeonpea cultivars ‘Asha’ and ‘Bahar’, respectively, to observe the cultivar-specific response to *P*. *fluorescens* OKC treatment. All 35 CcWRKY genes were up-regulated in ‘Asha’ whereas only 33 CcWRKY genes in ‘Bahar’ (*CcWRKYs 22* and 38 were down-regulated) in the *P*. *fluorescens* OKC treatment. Interestingly, 5 CcWRKYs (*CcWRKY*s *10*, *11*, *12*, *31*, and 37) in ‘Asha’ and 3 CcWRKYs (*CcWRKY*s *24*, *26*, and 43) in ‘Bahar’ showed more than 50 fold up-regulation. The results indicated that OKC induce the expression of all 35 selected CcWRKYs (Fig. 2, Supplementary Tables S5 and S6).

### Transcript profiling of the CcWRKY genes in response to combined stresses of *Fusarium udum* and NaCl

Transcript profiling of the WRKY genes was done in both the *F*. *udum* resistant and susceptible pigeonpea cultivars ‘Asha’ and ‘Bahar’, respectively, to observe the cultivar-specific response to the combined challenges of *F*. *udum* and NaCl. In most of the earlier studies WRKY genes were analyzed only either in biotic or abiotic stress conditions. In the present study we analyzed the combined effect of the pathogen and NaCl stress in a single treatment on the CcWRKY genes. Transcript accumulation of the CcWRKY genes showed differential patterns in both ‘Asha’ and ‘Bahar’ cultivars. In ‘Asha’ only *CcWRKY25* was down-regulated but the rest of the CcWRKY genes were up-regulated with highest fold change increase in *CcWRKY12*. Similarly, CcWRKY genes in ‘Bahar’ showed down-regulation of 10 *CcWRKY*s (*CcWRKY*s *21*, *22*, *31*, *32*, *33*, *34*, *36*, *37*, *39*, and 42) compared to only one in ‘Asha’. Interestingly, 5 CcWRKYs (*CcWRKY*s *30*, *35*, *40*, *41*, and 43) which were down-regulated in the individual NaCl treatment were slightly up-regulated in the combined application of the pathogen and NaCl with the highest fold change in *CcWRKY30*. Additionally, out of the 17 CcWRKYs that were observed to be better NaCl responsive compared to *F*. *udum* in the single application of NaCl (as listed in 2.6.2), 11 (*CcWRKYs 1*, *10*, *11*, *12*, *13*, *14*, *20*, *26*, *38*, *40* and 43) were identified to be dual responsive following combined application of both the stresses. This indicates dual role of these 11 CcWRKYs in addressing both *F*. *udum* and NaCl challenges (Fig. 2, Supplementary Tables S5 and S6).

### Transcript profiling of the CcWRKY genes in response to *Fusarium udum* and *Pseudomonas fluorescens* OKC

Transcript profiling of the WRKY genes was done in both the *F*. *udum* resistant and susceptible pigeonpea cultivars ‘Asha’ and ‘Bahar’, respectively, to observe the cultivar-specific response to *P*. *fluorescens* OKC treatment followed by *F*. *udum* challenge. In the resistant cultivar ‘Asha’ only *CcWRKY25* was down-regulated while all other CcWRKY genes were up-regulated. *CcWRKY25* also did not show true responsiveness to *F*. *udum* without OKC. Interestingly, most CcWRKYs which did not respond well to *F*. *udum* challenge without OKC were highly up-regulated in presence of OKC. *CcWRKY*s *3*, *10*, *11*, *12*, *13*, *14*, *15*, *20*, *21*, *23*, *24*, *26*, *27*, *28*, *29*, *30*, *31*, *32*, *33*, *34*, *35*, *36*, *37*, *38*, *39*, *40*, *41*, and 42 showed more fold changes compared to pathogen stress alone without OKC treatment with highest fold change in *CcWRKY39* while other CcWRKY genes namely *CcWRKY*s *1*, *4*, *5*, *7*, *22*, and 43 showed lower fold changes. In contrast, in the susceptible cultivar ‘Bahar’ differential fold changes were observed compared to the ‘Asha’ cultivar. *CcWRKY*s *1*, *3*, *5*, *7*, *12*, *13*, *15*, *20*, *24*, *25*, *29*, and 38 showed higher fold changes than pathogen stress alone while other *CcWRKY*s *11*, *14*, *23*, *27*, *28*, *35*, and 41 showed lower fold changes and *CcWRKY*s *21*, *30*, *31*, *32*, and 40, were down-regulated. There were some CcWRKY genes (*CcWRKY*s *22*, *33*, *34*, *36*, *37*, *39*, and 42) that were down-regulated in both pathogen and pathogen with OKC treatments in ‘Bahar’ cultivar. The results indicated minimal role of the selected CcWRKYs in the *F*. *udum* susceptible pigeonpea cultivar ‘Bahar’ except *CcWRKY*s *11*, *13*, and 14 where the role of OKC is established in sensitizing the genes (Fig. 2, Supplementary Tables S5 and S6).

### Transcript profiling of the CcWRKY genes in response to NaCl and *Pseudomonas fluorescens* OKC

Transcript profiling of the WRKY genes was done in both the *F*. *udum* resistant and susceptible pigeonpea cultivars ‘Asha’ and ‘Bahar’, respectively, to observe the cultivar-specific response to *P*. *fluorescens* OKC treatment followed by NaCl challenge. In ‘Asha’, 22 CcWRKY genes viz., *CcWRKY*s *1*, *11*, *13*, *20*, *21*, *22*, *24*, *26*, *27*, *28*, *29*, *30*, *31*, *32*, *33*, *34*, *35*, *36*, *37*, *40*, *41*, and 43 were up-regulated under NaCl response in which higher fold changes were observed when OKC applied as seed priming. Interestingly *CcWRKY25* that was down-regulated in NaCl treatment was up-regulated in NaCl treatment combined with OKC treatment. Some other *CcWRKY*s *3*, *4*, *5*, *7*, *10*, *12*, *14*, *15*, *23*, *38*, *39*, and 42 that were up-regulated under NaCl stress were also up-regulated in the combined treatment with OKC but relatively in lower folds compared to NaCl treatment alone. In ‘Bahar’ also a similar trend was seen in OKC treatments as in ‘Asha’. The CcWRKY genes (*CcWRKY*s *1*, *3*, *4*, *5*, *12*, *15*, *16*, *23*, *25*, *26*, *27*, *28*, and 29) that were up-regulated in NaCl treatment without OKC found increase in fold changes under NaCl treatment combined with OKC. Some *CcWRKY*s (*CcWRKY*s *30*, *35*, *36*, and 43) that were down-regulated in NaCl treatment alone were up-regulated in NaCl treatment combined with OKC. In *CcWRKY*s *7*, *10*, *11*, *13*, *14*, and 24 less fold change was observed during their up-regulation in NaCl treatment with OKC compared to NaCl treatment alone. There were other *CcWRKY*s (*CcWRKY*s *21*, *22*, *31*, *32*, *33*, *34*, *37*, *39*, *40*, *41*, and 42) which were down-regulated in both NaCl alone and NaCl with OKC treatment indicating their non-involvement in NaCl stress mediation. Only *CcWRKY38* which was up-regulated in NaCl treatment without OKC was down-regulated in NaCl with OKC treatment (Fig. 2, Supplementary Tables S5 and S6).

### Transcript profiling of the CcWRKY genes in response to combined inoculation of NaCl and *Fusarium udum* with OKC

Transcript profiling of the WRKY genes was done in both the *F*. *udum* resistant and susceptible pigeonpea cultivars ‘Asha’ and ‘Bahar’, respectively, to observe the cultivar-specific response to *P*. *fluorescens* OKC treatment followed by combined challenges of *F*. *udum* and NaCl. In ‘Asha’ all CcWRKY genes were up-regulated in different folds except *CcWRKY40*. Among those, 11 CcWRKY genes (*CcWRKY*s *7*, *10*, *14*, *15*, *22*, *23*, *28*, *29*, *30*, *32*, and 39) were up-regulated in higher folds whereas 7 CcWRKYs (*CcWRKY*s *11*, *12*, *21*, *26*, *33*, *34*, and 37) up-regulated in lower folds in presence of OKC. *CcWRKY*25 that was down-regulated without OKC was up-regulated in presence of OKC whereas *CcWRKY40* was down-regulated. Similarly, in ‘Bahar’ only 4 CcWRKY genes i.e. *CcWRKY*s *3*, *4*, *7*, and *25* showed higher fold change in presence of OKC while in 4 (*CcWRKY*s *1*, *12*, *13*, and 26) the fold change was lowered. *CcWRKY*s *21*, *22*, *31*, *32*, *33*, *34*, *36*, *37*, *39*, and 42 were down-regulated with or without OKC while *CcWRKY*s *30*, *35*, *38*, *40*, and 43 were down-regulated in presence of OKC (Fig. 2, Supplementary Tables S5 and S6). The results clearly indicated cross-talk between the *F*. *udum* and NaCl challenges.

## Discussion

Cells regulate gene expression through control of the transcriptional mechanism. WRKY proteins are also considered as transcriptional regulators. Proper annotation of genes of a gene family is important and essentially required for their functional studies. We identified and categorized 97 gene sequences as members of the WRKY family in pigeonpea (*Cajanus cajan*) and *in silico* functional analysis was done using BLAST as a tool to search the NCBI and TAIR databases. Results indicated that majority of the WRKY proteins in pigeonpea showed similarity with biotic and abiotic stress-related WRKY proteins of *Arabidopsis thaliana*. It made us to propose that CcWRKY proteins are also involved in resistance activities against various biotic and abiotic stresses as in *A*. *thaliana*.

The phylogenetic relationship study used in the present work allowed division of the pigeonpea WRKY genes into the three previously reported groups^9^. Though the WRKYGQK sequence was found highly conserved among pigeonpea WRKY proteins (Supplementary Fig. SF2), variations are observed in 9 CcWRKY genes. Previous studies also showed that GmWRKY6 and GmWRKY21 in soybean have the variant sequence WRKYGKK rather than the conserved WRKYGQK motif sequence^25^. Minor variations at this particular region was also reported earlier in Arabidopsis, sunflower, barley, rice, tobacco, and canola^19,26–30^. Variation in the conserved protein sequences may affect adversely resulting in termination or decreasing the capacity of WRKY proteins in binding with the signature W-box element^3^. It is quite possible that the WRKY proteins without the conserved WRKYGQK motif may bind to a different site in the target genes^28^ and play a different role^27^. For example, soybean WRKY protein GmWRKY6 and tobacco WRKY protein NtWRKY12 can bind to WK-box (TTTTCCAC) instead of binding to the canonical W-box element^25,28^. In the present study also, we observed that some CcWRKYs showed variations in the zinc finger motif in a similar way with the three VvWRKY proteins of *Vitis vinifera*^31^. However, the functionality of such variations in the zinc-finger like motif remains to be elucidated. In addition to the conserved WRKY domain, other motifs which are observed as conserved sequence could be important for different functions of the WRKY proteins. Up-regulation of the *CcWRKY7* gene more than 50 fold under NaCl stress might be due to the presence of the extra motif 24 in this gene.

In field conditions, crop plants face variety of biotic and abiotic stresses and thereby remain under continuous threat. Plants adapt to these kinds of situations through reprogramming of their inherent metabolic pathways via differential expression of genes. WRKY gene family can respond to these situations and act as activators or repressors of certain genes of the crop species. In the current study, transcript accumulation patterns of 35 *CcWRKY* genes under the two stresses (*F*. *udum* and NaCl) with or without application of *Pseudomonas fluorescens* OKC in two pigeonpea cultivars revealed distinct variations in different treatments. Responsiveness of these selected *WRKY* genes to *F*. *udum* challenge in the resistant cultivar ‘Asha’ confirms their selection and validates their functionality. Significant up-regulation of most of the 35 selected biotic stress-responsive *CcWRKY* genes in the resistant cultivar ‘Asha’ endorses their responsiveness to *F*. *udum* stress and thereby, contributing to resisting progress of the pathogen in pigeonpea. Further, relatively high responsiveness of 4 *CcWRKY*s (*CcWRKYs 12*, *13*, *31* and 41) to *F*. *udum* stress in both the cultivars can be attributed to their involvement in the initial response by pigeonpea to *F*. *udum*. Interestingly, 4 *CcWRKY*s (*CcWRKYs 15*, *21*, *24* and 36) that did not respond highly to *F*. *udum* stress, responded very strongly when the plants were bio-primed with the *P*. *fluorescens* OKC strain. Significance of PGPR strains in reducing NaCl stress in pulses had been recorded earlier^32,33^. However, the mechanisms underlying thereof particularly the role of WRKY transcription factors have not been worked out so far. The results thus demonstrate significance of application of PGPR strains such as OKC in pigeonpea for enhancing resistance against biotic stresses such as *F*. *udum* through enhanced activation of CcWRKYs. Additionally, the role of OKC in enhancing transcript accumulation in some other CcWRKYs (*CcWRKYs 25*, *30* and 35) which were otherwise responded lowly to the lone NaCl stress indicated its worth in enhancing salt tolerance in pigeonpea as well. However, the role of OKC was not very evident in enhancing transcript levels of the selected CcWRKYs in combined application of both the stresses (*F*. *udum* and NaCl) compared to its ability to enhance transcript levels of the same CcWRKYs under the same stresses when applied individually. The variations may be attributed to cross-talk of the signaling cascades operative under *F*. *udum* as well as NaCl stresses in pigeonpea. Additionally, the use of microbial consortium may be another option to address dual stresses of different kinds^34^.

Although, different WRKY genes respond to different stresses some of the WRKY genes are also reported to respond to combined biotic and abiotic stresses^35^. In the current study, all 35 CcWRKY genes showed differential expression under salt stress, where the expression of all the genes were up-regulated except *CcWRKY25* in ‘Asha’ compared to ‘Bahar’ in which only 25 CcWRKYs genes were up-regulated and rest 10 were down-regulated. This variation in expression of the same genes in the susceptible cultivar ‘Bahar’ may be attributed to lack or absence of some common factors (genes) that govern both biotic and abiotic stresses in the resistant pigeonpea cultivar ‘Asha’. However, it was interesting to note that among the 35 selected biotic stress-responsive CcWRKY genes 26 were responsive to *F*. *udum*, 17 were NaCl responsive and most significantly 11 were dual responsive to both *F*. *udum* and NaCl stresses in both the pigeonpea cultivars. This shows the role of the 11 CcWRKY genes in mitigating both biotic and abiotic stresses probably in most of the cultivated pigeonpea cultivars. Differential expression patterns of the same WRKY genes were also recorded in 54 OsWRKY from rice and 26 GmWRKY from soybean under abiotic stresses^36^. Further, it is quite likely that the highly induced CcWRKY genes might also play an important role against other abiotic stresses as it was shown in case of *BcWRKY46* which is a cold-inducible gene from Pak-choi also enhances tolerance to salt and dehydration stresses in transgenic tobacco^37^ and in 23 CsWRKY genes from *Cucumis sativus* that expressed differentially in response to at least one abiotic stress such as cold, drought, or salinity^13^. From the current study, it can be concluded that the 35 biotic stress responsive *CcWRKY* genes identified in pigeonpea, based on the homologous genes from the representative plant species *A*. *thaliana*, responded well to *F*. *udum* stress and thereby confirm their selection as biotic stress responsive CcWRKYs. However, among the selected 35 CcWRKYs, 26 responded very highly to *F*. *udum*, 17 responded highly to NaCl compared to *F*. *udum* and 11 were dual responsive to both *F*. *udum* and NaCl. The study thus signifies the role of the biotic stress responsive CcWRKYs in mitigating *F*. *udum* and NaCl stresses in pigeonpea.

## Materials and Methods

### Plant materials, pathogen and bio-agent culture

Pigeonpea varieties ‘Asha’ (Fusarium wilt resistant) and ‘Bahar’ (Fusarium wilt susceptible) and the wilt pathogen *Fusarium udum* strain WSP-V2 were obtained from Indian Institute of Pulses Research, Kanpur, Uttar Pradesh, India. *Pseudomonas fluorescens* OKC (Accession numbers JN128891) was used as a PGPR and biocontrol agent having antagonistic properties against *F*. *udum*.

### Biopriming and stress inoculation

Seeds of cultivars ‘Asha’ and ‘Bahar’ were bio-primed with *Pseudomonas fluorescens* OKC according to Yadav *et al*.^38^. Briefly, cell suspension of OKC was adjusted to 1.6 × 10^8^ cfu mL^−1^ before seed biopriming. Seed were bio-primed for 2 h, dried under shed at room temperature and the primed seeds were sown in sterilized garden soil mixed with 10% vermiculite. Five replicates of each treatment were maintained. After 21 days of sowing, plants were inoculated with conidial suspension (2 × 10^7^ conidia/mL) of *Fusarium udum* and 200 mM NaCl either alone or in combination. Conidial suspensions and NaCl solutions were prepared in sterilized distilled water. Root samples were collected 24 h after pathogen and salt inoculation for quantification of transcripts of target genes by quantitative RT-PCR. The treatments applied were: (i) *Fusarium udum*, (ii) NaCl (200 mM), (iii) *Fusarium udum* + NaCl and (iv) untreated control. One set of all 4 treatments were maintained separately without seed bio-priming with OKC.

### RNA isolation, cDNA synthesis and qRT-PCR analysis

Total RNA was extracted and purified from 200 mg of homogenized root tissue thrice from each sample with some modifications and quantified using a Nano-Drop 2000 UV Vis spectrophotometer (Thermo Scientific, Wilmington, DE, USA)^39^. Approximately 3 μg total RNA was treated using RNase-free DNase I at 37 °C to remove the remaining genomic DNA. cDNA was prepared using oligo (dT) primers and reverse transcriptase enzyme according to Sambrook and Russell^40^. cDNAs were used as templates for semi-quantitative and quantitative RT-PCR.

### Sequence analysis and classification of CcWRKY genes

*Cajanus cajan* WRKY genomic and protein sequences were retrieved from Plant Transcription Factor database^41^ and analysis were completed to confirm the presence of the WRKY domain using the SMART program^42^. Additionally, primary structure analysis of CcWRKY proteins such as length, molecular weight, isoelectric point, total number of atoms extinction coefficients, instability index, aliphatic index, grand average of hydropathicity was completed using the ExPasy website (http://au.expasy.org/tools/pi_tool.html), whereas protein homology study of WRKY proteins was done using the Basic Local Alignment Search Tool (BLAST). Since detailed study of WRKY family was done in Arabidopsis^8^, phylogenetic tree was constructed based on the alignment of WRKY domains from pigeonpea and *Arabidopsis* to evaluate the phylogenetic relationships and classified them into different groups^8^.

### Motif analysis and phylogenetic relationship

WRKY proteins were also used to detect the conserved motifs using MEME (http://meme.nbcr.net/meme/cgibin/meme.cgi) with parameters such as number of repetitions: any; maximum number of motifs: 25; and the optimum motif widths: 6–200 amino acid residues. Software MEGA was used to prepare the phylogenetic tree and neighbor-joining method was adopted by 1,000 bootstrap replications^8^. Functional analysis of motifs was done through NCBI protein blast search within the protein databank of plant.

### Primer designing and transcript profiling of CcWRKY genes

cDNA sequences of the candidate *CcWRKY* genes were retrieved from GenBank and primers were designed by using the online software Primer-3^43^ (Supplementary Table S7). In order to prevent off-target amplification, the most homologous sequences of the WRKY TFs were identified through BLAST search in the NCBI database. Sequence alignment was done using Clustal Omega, and the software suggested primers were compared to the alignments to select the primers with the least homology to off target sequences. Transcript profiling of the selected CcWRKY transcription factors was carried out using qRT-PCR according to the modified protocol of Marone *et al*.^44^ in three technical replicates per sample. Eva Green SYBR Green Supermix Kit (Bio-Rad) was used in a iQ5 Real-Time PCR Detection System (Bio-Rad Laboratories, Munchen, Germany) for qRT-PCR. We used gene-specific primers at a final concentration of 10pmol/µl and transcript levels of each mRNA were determined and normalized with the level of internal control. The PCR condition involved denaturation at 95 °C for 2 min, 40 repeats at 95 °C for 30 s, 60 °C for 30 s, and 72 °C for 30 s. Actin (Accession No. XM_020367686.1) and β-tubulin (Accession No. XM_020353202.1) were used as internal controls. Data normalization was done with mean CT values of the endogenous genes Actin and β-tubulin using the 2^−ΔΔCT^ method^45^. Fold accumulation of transcripts was compared by using the mean of the CT values of the three technical replicates from each five biological replications with control.

## Conclusions

WRKY genes family is known to be responsive to both biotic and abiotic stresses. We analyzed the transcript accumulation patterns of 35 biotic stress responsive CcWRKY genes against biotic (*Fusarium udum*) and abiotic (NaCl) stresses alone and in combination treatments in the *F*. *udum* resistant and susceptible pigeonpea cultivars ‘Asha’ and ‘Bahar’. A comparative expression profile of highly and lowly up-regulated *CcWRKY*s is presented in Supplementary Table S8 based on their responses in the resistant cultivar ‘Asha’ under the two individual stresses of *F*. *udum* and NaCl. We observed that in ‘Asha’, most of the selected CcWRKYs were up-regulated in all treatments except *CcWRKY25* which was down-regulated. Similarly, *CcWRKY40* was up-regulated in all treatments except in two treatments viz., *F*. *udum* and NaCl + OKC. However, differential expression patterns of CcWRKY genes were observed in ‘Bahar’ compared to ‘Asha’. In ‘Bahar’ eighteen CcWRKY genes (*CcWRKY*s *1*, *2*, *4*, *5*, *7*, *10*, *11*, *12*, *13*, *14*, *15*, *23*, *24*, *25*, *26*, *27*, *28*,and 29) were up-regulated in all treatments while rest of the CcWRKY genes (*CcWRKY*s *20*, *21*, *30*, *31*, *32*, *33*, *34*, *36*, *37*, *38*, *39*, *40*, *41*, *42*, and 43) were differentially expressed in different treatments either lowly up-regulated or down-regulated. Interestingly, *CcWRKY22* was down-regulated in all treatments in ‘Bahar’. To conclude, 26 CcWRKYs were observed to be highly *F*. *udum* responsive and 9 least responsive, and among the 35 CcWRKY genes selected 17 are better NaCl responsive compared to *F*. *udum* and 11 are dual responsive to both *F*. *udum* and NaCl. The PGPR strain *P*. *fluorescens* OKC proved to play a role in stimulating CcWRKY-mediated stress responsiveness in pigeonpea in individual stresses of *F*. *udum* and NaCl compared to their combined stresses.

## Supplementary information

Supplementary Figures and tables
