# Supplementary material for: Identification, characterization and expression profiles of Fusarium udum stress-responsive WRKY transcription factors in Cajanus cajan under the influence of NaCl stress and Pseudomonas fluorescens OKC
Source: Sci Rep. 2019 Oct 4;9:14344. doi: 10.1038/s41598-019-50696-x (PMC6778267; doi:10.1038/s41598-019-50696-x)
Supplement: Supplementary file 1 — Supplementary Figures and tables [file 41598_2019_50696_MOESM1_ESM.pdf]

Identification, characterization and expression profiles of *Fusarium udum* stress-responsive *WRKY* transcription factors in *Cajanus cajan* under influence of NaCl stress and *Pseudomonas fluorescens* OKC

Gagan Kumar[1], Raina Bajpai[1], Ankita Sarkar[1], Raj Kumar Mishra[2], Vijay Kumar Gupta[3], Harikesh B. Singh[1] and Birinchi K. Sarma[1]*

Table S1: CcWRKYs detail according to their accession number

Accession no.	CcWRKY genes	Accession no.	CcWRKY genes	Accession no.	CcWRKY genes	Accession no.	CcWRKY genes
C.cajan_24537	*CcWRKY1*	C.cajan_26666	*CcWRKY26*	C.cajan_05913	*CcWRKY51*	C.cajan_26986	*CcWRKY76*
C.cajan_29780	*CcWRKY2*	C.cajan_02335	*CcWRKY27*	C.cajan_07333	*CcWRKY52*	C.cajan_27136	*CcWRKY77*
C.cajan_29457	*CcWRKY3*	C.cajan_28176	*CcWRKY28*	C.cajan_08309	*CcWRKY53*	C.cajan_27183	*CcWRKY78*
C.cajan_13648	*CcWRKY4*	C.cajan_16563	*CcWRKY29*	C.cajan_08356	*CcWRKY54*	C.cajan_27628	*CcWRKY79*
C.cajan_20775	*CcWRKY5*	C.cajan_25148	*CcWRKY30*	C.cajan_08357	*CcWRKY55*	C.cajan_27637	*CcWRKY80*
C.cajan_29116	*CcWRKY6*	C.cajan_25149	*CcWRKY31*	C.cajan_08458	*CcWRKY56*	C.cajan_28168	*CcWRKY81*
C.cajan_13299	*CcWRKY7*	C.cajan_04816	*CcWRKY32*	C.cajan_08473	*CcWRKY57*	C.cajan_28399	*CcWRKY82*
C.cajan_13689	*CcWRKY8*	C.cajan_41239	*CcWRKY33*	C.cajan_09788	*CcWRKY58*	C.cajan_29119	*CcWRKY83*
C.cajan_23649	*CcWRKY9*	C.cajan_05144	*CcWRKY34*	C.cajan_10884	*CcWRKY59*	C.cajan_30708	*CcWRKY84*
C.cajan_00060	*CcWRKY10*	C.cajan_05149	*CcWRKY35*	C.cajan_11749	*CcWRKY60*	C.cajan_32192	*CcWRKY85*
C.cajan_26275	*CcWRKY11*	C.cajan_04448	*CcWRKY36*	C.cajan_13633	*CcWRKY61*	C.cajan_32591	*CcWRKY86*
C.cajan_08231	*CcWRKY12*	C.cajan_16799	*CcWRKY37*	C.cajan_14327	*CcWRKY62*	C.cajan_32672	*CcWRKY87*
C.cajan_41159	*CcWRKY13*	C.cajan_27330	*CcWRKY38*	C.cajan_14927	*CcWRKY63*	C.cajan_35643	*CcWRKY88*
C.cajan_03812	*CcWRKY14*	C.cajan_35299	*CcWRKY39*	C.cajan_15948	*CcWRKY64*	C.cajan_37436	*CcWRKY89*
C.cajan_10680	*CcWRKY15*	C.cajan_17992	*CcWRKY40*	C.cajan_18219	*CcWRKY65*	C.cajan_39263	*CcWRKY90*
C.cajan_18758	*CcWRKY16*	C.cajan_04900	*CcWRKY41*	C.cajan_20299	*CcWRKY66*	C.cajan_39557	*CcWRKY91*
C.cajan_05407	*CcWRKY17*	C.cajan_05822	*CcWRKY42*	C.cajan_20860	*CcWRKY67*	C.cajan_40968	*CcWRKY92*
C.cajan_19828	*CcWRKY18*	C.cajan_42797	*CcWRKY43*	C.cajan_23223	*CcWRKY68*	C.cajan_47800	*CcWRKY93*
C.cajan_28195	*CcWRKY19*	C.cajan_00971	*CcWRKY44*	C.cajan_23650	*CcWRKY69*	C.cajan_43040	*CcWRKY94*
C.cajan_29944	*CcWRKY20*	C.cajan_01682	*CcWRKY45*	C.cajan_23812	*CcWRKY70*	C.cajan_44344	*CcWRKY95*
C.cajan_36768	*CcWRKY21*	C.cajan_02255	*CcWRKY46*	C.cajan_25091	*CcWRKY71*	C.cajan_44345	*CcWRKY96*
C.cajan_41592	*CcWRKY22*	C.cajan_03945	*CcWRKY47*	C.cajan_25490	*CcWRKY72*	C.cajan_48561	*CcWRKY97*
C.cajan_08305	*CcWRKY23*	C.cajan_04594	*CcWRKY48*	C.cajan_25681	*CcWRKY73*		
C.cajan_40644	*CcWRKY24*	C.cajan_05139	*CcWRKY49*	C.cajan_26080	*CcWRKY74*		
C.cajan_43923	*CcWRKY25*	C.cajan_05401	*CcWRKY50*	C.cajan_26985	*CcWRKY75*		

Table S2: In silico functional analysis of CcWRKYs

PTFDB ID	PRJNA (NCBI)	Accession no.	Best match in *Arabidopsis thaliana* (TAIR)	Accession no.	Function (TAIR)
C.cajan_00060	CcWRKY40	KYP53896.1	ATWRKY40	AT1G80840.1	defense response to bacterium, defense response to fungus, negative regulation of transcription, DNA-templated, regulation of defense response, regulation of defense response to virus by host, regulation of transcription, DNA-templated, response to chitin, response to molecule of bacterial origin, response to salicylic acid, response to wounding, transcription, DNA-templated
C.cajan_00971	CcWRKY39	KYP54799.1	ATWRKY39	AT3G04670.1	regulation of transcription, DNA-templated, transcription, DNA-templated
C.cajan_01682	CcWRKY61	XP_020225151.1	ATWRKY61	AT1G18860.1	regulation of transcription, DNA-templated, transcription, DNA-templated
C.cajan_02255	CcWRKY46	XP_020224984.1	ATWRKY46	AT2G46400.1	lateral root development, regulation of transcription, DNA-templated, response to chitin, transcription, DNA-templated
C.cajan_02335	CcWRKY23	XP_020226824.1	ATWRKY23	AT2G47260.1	regulation of transcription, DNA-templated, response to auxin, response to nematode, transcription, DNA-templated
C.cajan_03812	CcWRKY42	KYP57628.1	ATWRKY42	AT4G04450.1	negative regulation of transcription, DNA-templated, regulation of transcription, DNA-templated, transcription, DNA-templated
C.cajan_03945	CcWRKY42	KYP57761.1	ATWRKY42	AT4G04450.1	negative regulation of transcription, DNA-templated, regulation of transcription, DNA-templated, transcription, DNA-templated
C.cajan_04448	CcWRKY6	KYP57761.1	ATWRKY6	AT1G62300.1	cellular response to boron-containing substance deprivation, cellular response to phosphate starvation, ethylene-activated signaling pathway, negative regulation of transcription, DNA-templated, regulation of transcription, DNA-templated, response to chitin, transcription, DNA-templated.
C.cajan_04594	CcWRKY41	KYP72128.1	ATWRKY41	AT4G11070.1	regulation of transcription, DNA-templated, transcription, DNA-templated
C.cajan_04816	CcWRKY11	XP_020239587.1	ATWRKY11	AT4G31550.1	defense response to bacterium, regulation of transcription, DNA-templated, response to chitin, transcription, DNA-templated
C.cajan_04900	CcWRKY3	KYP72432.1	ATWRKY3	AT2G03340.1	regulation of transcription, DNA-templated, transcription,

					DNA-templated
C.cajan_05139	CcWRKY70	KYP72670.1	ATWRKY70	AT3G56400.1	defense response to bacterium, defense response to fungus, induced systemic resistance, jasmonic acid mediated signaling pathway, negative regulation of leaf senescence, negative regulation of transcription, DNA-templated, regulation of defense response, regulation of transcription, DNA-templated, response to chitin, response to jasmonic acid, response to salicylic acid, systemic acquired resistance, salicylic acid mediated signaling pathway, transcription, DNA-templated
C.cajan_05144	CcWRKY70	KYP72675.1	ATWRKY70	AT3G56400.1	defense response to bacterium, defense response to fungus, induced systemic resistance, jasmonic acid mediated signaling pathway, negative regulation of leaf senescence, negative regulation of transcription, DNA-templated, regulation of defense response, regulation of transcription, DNA-templated, response to chitin, response to jasmonic acid, response to salicylic acid, systemic acquired resistance, salicylic acid mediated signaling pathway, transcription, DNA-templated
C.cajan_05149	CcWRKY70	KYP72680.1	ATWRKY70	AT3G56400.1	defense response to bacterium, defense response to fungus, induced systemic resistance, jasmonic acid mediated signaling pathway, negative regulation of leaf senescence, negative regulation of transcription, DNA-templated, regulation of defense response, regulation of transcription, DNA-templated, response to chitin, response to jasmonic acid, response to salicylic acid, systemic acquired resistance, salicylic acid mediated signaling pathway, transcription, DNA-templated.
C.cajan_05401	CcWRKY34	KYP72930.1	ATWRKY34	AT4G26440.1	pollen development, pollen germination, regulation of transcription, DNA-templated, response to cold, transcription, DNA-templated
C.cajan_05407	CcWRKY24	XP_020226257.1	ATWRKY33	AT2G38470.1	camalexin biosynthetic process, cellular heat acclimation, cellular response to heat, defense response to bacterium, defense response to fungus, positive regulation of autophagy, regulation of transcription, DNA-templated, response to chitin, response to cold, response to heat, response to osmotic stress, response to salt stress, response to water deprivation, transcription, DNA-templated
C.cajan_05822	CcWRKY71	XP_020204261.1	ATWRKY28	AT4G18170.1	regulation of transcription, DNA-templated, transcription, DNA-templated

C.cajan_05913	CcWRKY6	KYP73437.1	ATWRKY6	AT1G62300.1	cellular response to boron-containing substance deprivation, cellular response to phosphate starvation, ethylene-activated signaling pathway, negative regulation of transcription, DNA-templated, regulation of transcription, DNA-templated, response to chitin, transcription, DNA-templated
C.cajan_07333	CcWRKY27	XP_020210484.1	ATWRKY27	AT5G52830.1	defense response to bacterium, negative regulation of transcription, DNA-templated, nitric oxide mediated signal transduction, regulation of transcription, DNA-templated, response to gibberellin, transcription, DNA-templated
C.cajan_08231	CcWRKY72	KYP69288.1	ATWRKY72	AT5G15130.1	defense response, regulation of transcription, DNA-templated, transcription, DNA-templated
C.cajan_08305	CcWRKY51	KYP69362.1	ATWRKY51	AT5G64810.1	defense response to bacterium, defense response to fungus, jasmonic acid mediated signaling pathway, regulation of transcription, DNA-templated, transcription, DNA-templated
C.cajan_08309	CcWRKY13	XP_020213068.1	ATWRKY13	AT4G39410.1	positive regulation of sclerenchyma cell differentiation, positive regulation of transcription, DNA-templated, regulation of lignin biosynthetic process, regulation of transcription, DNA-templated, transcription, DNA-templated
C.cajan_08356	CcWRKY13	KYP69413.1	ATWRKY13	AT4G39410.1	positive regulation of sclerenchyma cell differentiation, positive regulation of transcription, DNA-templated, regulation of lignin biosynthetic process, regulation of transcription, DNA-templated, transcription, DNA-templated
C.cajan_08357	CcWRKY13	KYP69413.1	ATWRKY13	AT4G39410.1	positive regulation of sclerenchyma cell differentiation, positive regulation of transcription, DNA-templated, regulation of lignin biosynthetic process, regulation of transcription, DNA-templated, transcription, DNA-templated
C.cajan_08458	CcWRKY75	KYP69515.1	ATWRKY75	AT5G13080.1	atrichoblast differentiation, lateral root development, negative regulation of transcription by RNA polymerase II, regulation of DNA-templated transcription in response to stress, regulation of response to nutrient levels, regulation of transcription, DNA-templated, transcription, DNA-templated
C.cajan_08473	CcWRKY75	KYP69530.1	ATWRKY75	AT5G13080.1	atrichoblast differentiation, lateral root development, negative regulation of transcription by RNA polymerase II, regulation of DNA-templated transcription in response to stress, regulation of response to nutrient levels,

					regulation of transcription, DNA-templated, transcription, DNA-templated
C.cajan_09788	CcWRKY44	XP_020212254.1	ATWRKY44	AT2G37260.1	epidermal cell fate specification, regulation of transcription, DNA-templated, seed coat development, transcription, DNA-templated
C.cajan_10680	CcWRKY42	KYP71720.1	ATWRKY42	AT4G04450.1	negative regulation of transcription, DNA-templated, regulation of transcription, DNA-templated, transcription, DNA-templated
C.cajan_10884	CcWRKY30	XP_020211127.1	ATWRKY41	AT4G11070.1	regulation of transcription, DNA-templated, transcription, DNA-templated
C.cajan_11749	CcWRKY72	KYP65836.1	ATWRKY72	AT5G15130.1	defense response, regulation of transcription, DNA-templated, transcription, DNA-templated
C.cajan_13299	CcWRKY41	XP_020217757.1	ATWRKY41	AT4G11070.1	regulation of transcription, DNA-templated, transcription, DNA-templated
C.cajan_13633	CcWRKY53	XP_020223272.1	ATWRKY41	AT4G11070.1	regulation of transcription, DNA-templated, transcription, DNA-templated
C.cajan_13648	CcWRKY29	KYP58649.1	ATWRKY29	AT4G23550.1	defense response, regulation of transcription, DNA-templated, response to chitin, transcription, DNA-templated
C.cajan_13689	CcWRKY41	XP_020223510.1	ATWRKY41	AT4G11070.1	regulation of transcription, DNA-templated, transcription, DNA-templated
C.cajan_14327	CcWRKY11	XP_020223656.1	ATWRKY11	AT4G31550.1	defense response to bacterium, regulation of transcription, DNA-templated, response to chitin, transcription, DNA-templated
C.cajan_14927	CcWRKY2	KYP59917.1	ATWRKY2	AT5G56270.1	establishment of cell polarity, longitudinal axis specification, pollen development, regulation of transcription, DNA-templated, transcription, DNA-templated
C.cajan_15948	CcWRKY27	KYP61901.1	ATWRKY27	AT5G52830.1	defense response to bacterium, negative regulation of transcription, DNA-templated, nitric oxide mediated signal transduction, regulation of transcription, DNA-templated, response to gibberellin, transcription, DNA-templated
C.cajan_16563	CcWRKY48	KYP62511.1	ATWRKY48	AT5G49520.1	defense response to bacterium, positive regulation of transcription, DNA-templated, regulation of transcription, DNA-templated, response to bacterium, response to chitin, transcription, DNA-templated
C.cajan_16799	CcWRKY6	KYP62746.1	ATWRKY6	AT1G62300.1	cellular response to boron-containing substance deprivation, cellular response to phosphate starvation, ethylene-activated signaling pathway, negative regulation of transcription, DNA-templated, regulation of transcription, DNA-templated, response to

					chitin, transcription, DNA-templated.
C.cajan_17992	CcWRKY4	KYP63936.1	ATWRKY4	AT1G13960.2	defense response, negative regulation of defense response to bacterium, regulation of defense response to fungus, regulation of transcription, DNA-templated, response to ethylene, response to jasmonic acid, response to salicylic acid, transcription, DNA-templated
C.cajan_18219	CcWRKY2	KYP64163.1	ATWRKY2	AT5G56270.1	establishment of cell polarity, longitudinal axis specification, pollen development, regulation of transcription, DNA-templated, transcription, DNA-templated
C.cajan_18758	CcWRKY3	KYP64700.1	ATWRKY4	AT1G13960	defense response, negative regulation of defense response to bacterium, regulation of defense response to fungus, regulation of transcription, DNA-templated, response to ethylene, response to jasmonic acid, response to salicylic acid, transcription, DNA-templated
C.cajan_19828	CcWRKY24	XP_020212386.1	ATWRKY33	AT2G38470.1	camalexin biosynthetic process, cellular heat acclimation, cellular response to heat, defense response to bacterium, defense response to fungus, positive regulation of autophagy, regulation of transcription, DNA-templated, response to chitin, response to cold, response to heat, response to osmotic stress, response to salt stress, response to water deprivation, transcription, DNA-templated
C.cajan_20299	CcWRKY70	XP_020218541.1	ATWRKY70	AT3G56400.1	defense response to bacterium, defense response to fungus, induced systemic resistance, jasmonic acid mediated signaling pathway, negative regulation of leaf senescence, negative regulation of transcription, DNA-templated, regulation of defense response, regulation of transcription, DNA-templated, response to chitin, response to jasmonic acid, response to salicylic acid, systemic acquired resistance, salicylic acid mediated signaling pathway, transcription, DNA-templated
C.cajan_20775	CcWRKY22	XP_020220494.1	ATWRKY22	AT4G01250.1	defense response, leaf senescence, regulation of transcription, DNA-templated, response to chitin, transcription, DNA-templated
C.cajan_20860	CcWRKY43	XP_020219778.1	ATWRKY56	AT1G64000.1	regulation of transcription, DNA-templated, transcription, DNA-templated
C.cajan_23223	CcWRKY13	XP_020215926.1	ATWRKY13	AT4G39410.1	positive regulation of sclerenchyma cell differentiation, positive regulation of transcription, DNA-templated, regulation of lignin biosynthetic process, regulation of transcription, DNA-templated, transcription, DNA-

					templated
C.cajan_23649	CcWRKY55	KYP53762.1	ATWRKY55	AT2G40740.1	regulation of transcription, DNA-templated, transcription, DNA-templated
C.cajan_23650	CcWRKY70	KYP53763.1	ATWRKY70	AT3G56400.1	defense response to bacterium, defense response to fungus, induced systemic resistance, jasmonic acid mediated signaling pathway, negative regulation of leaf senescence, negative regulation of transcription, DNA-templated, regulation of defense response, regulation of transcription, DNA-templated, response to chitin, response to jasmonic acid, response to salicylic acid, systemic acquired resistance, salicylic acid mediated signaling pathway, transcription, DNA-templated
C.cajan_23812	CcWRKY32	KYP53607.1	ATWRKY32	AT4G30935.1	regulation of transcription, DNA-templated, transcription, DNA-templated
C.cajan_24537	CcWRKY15	XP_020230359.1	ATWRKY15	AT2G23320.1	regulation of transcription, DNA-templated, response to chitin, transcription, DNA-templated
C.cajan_25091	CcWRKY51	XP_020230188.1	ATWRKY11	AT4G31550.1	defense response to bacterium, regulation of transcription, DNA-templated, response to chitin, transcription, DNA-templated
C.cajan_25148	CcWRKY23	KYP52616.1	ATWRKY23	AT2G47260.1	regulation of transcription, DNA-templated, response to auxin, response to nematode, transcription, DNA-templated
C.cajan_25149	CcWRKY48	XP_020230271.1	ATWRKY23	AT2G47260.1	regulation of transcription, DNA-templated, response to auxin, response to nematode, transcription, DNA-templated
C.cajan_25490	CcWRKY6	KYP52183.1	ATWRKY6	AT1G62300.1	cellular response to boron-containing substance deprivation, cellular response to phosphate starvation, ethylene-activated signaling pathway, negative regulation of transcription, DNA-templated, regulation of transcription, DNA-templated, response to chitin, transcription, DNA-templated.
C.cajan_25681	CcWRKY14	KYP52057.1	ATWRKY14	AT1G30650.1	regulation of transcription, DNA-templated, transcription, DNA-templated
C.cajan_26080	CcWRKY72	KYP50654.1	ATWRKY72	AT5G15130.1	defense response, regulation of transcription, DNA-templated, transcription, DNA-templated
C.cajan_26275	CcWRKY40	KYP50443.1	ATWRKY40	AT1G80840.1	defense response to bacterium, defense response to fungus, negative regulation of transcription, DNA-templated, regulation of defense response, regulation of defense response to virus by host, regulation of transcription, DNA-templated, response to chitin, response to molecule of bacterial origin, response to salicylic acid, response to wounding, transcription, DNA-

					templated
C.cajan_26666	CcWRKY28	XP_020232986.1	ATWRKY28	AT4G18170.1	regulation of transcription, DNA-templated, transcription, DNA-templated
C.cajan_26985	CcWRKY20	KYP49857.1	ATWRKY20	AT4G26640.2	positive regulation of transcription, DNA-templated, regulation of transcription, DNA-templated, response to 1-aminocyclopropane-1-carboxylic acid, response to wounding, transcription, DNA-templated
C.cajan_26986	CcWRKY20	KYP49858.1	ATWRKY20	AT4G26640.2	positive regulation of transcription, DNA-templated, regulation of transcription, DNA-templated, response to 1-aminocyclopropane-1-carboxylic acid, response to wounding, transcription, DNA-templated
C.cajan_27136	CcWRKY61	XP_020232011.1	ATWRKY72	AT5G15130.1	defense response, regulation of transcription, DNA-templated, transcription, DNA-templated
C.cajan_27183	CcWRKY1	KYP48145.1	ATWRKY1	AT2G04880.1	positive regulation of transcription, DNA-templated, regulation of transcription, DNA-templated, salicylic acid mediated signaling pathway, transcription, DNA-templated
C.cajan_27330	CcWRKY6	KYP48279.1	ATWRKY6	AT1G62300.1	cellular response to boron-containing substance deprivation, cellular response to phosphate starvation, ethylene-activated signaling pathway, negative regulation of transcription, DNA-templated, regulation of transcription, DNA-templated, response to chitin, transcription, DNA-templated.
C.cajan_27628	CcWRKY9	KYP47924.1	ATWRKY9	AT1G68150.1	regulation of transcription, DNA-templated, transcription, DNA-templated
C.cajan_27637	CcWRKY14	KYP47933.1	ATWRKY14	AT1G30650.1	regulation of transcription, DNA-templated, transcription, DNA-templated
C.cajan_28168	CcWRKY65	XP_020234857.1	ATWRKY35	AT2G34830.1	embryo development ending in seed dormancy, pollen development, regulation of transcription, DNA-templated, transcription, DNA-templated
C.cajan_28176	CcWRKY23	XP_020234847.1	ATWRKY23	AT2G47260.1	regulation of transcription, DNA-templated, response to auxin, response to nematode, transcription, DNA-templated
C.cajan_28195	CcWRKY33	KYP45283.1	ATWRKY33	AT2G38470.1	camalexin biosynthetic process, cellular heat acclimation, cellular response to heat, defense response to bacterium, defense response to fungus, positive regulation of autophagy, regulation of transcription, DNA-templated, response to chitin, response to cold, response to heat, response to osmotic stress, response to salt stress, response to water deprivation, transcription, DNA-templated
C.cajan_28399	CcWRKY39	KYP49226.1	ATWRKY39	AT3G04670.1	regulation of transcription, DNA-templated, transcription,

					DNA-templated
C.cajan_29116	CcWRKY22	KYP47600.1	ATWRKY27	AT5G52830.1	defense response to bacterium, negative regulation of transcription, DNA-templated, nitric oxide mediated signal transduction, regulation of transcription, DNA-templated, response to gibberellin, transcription, DNA-templated
C.cajan_29119	CcWRKY24	KYP47603.1	ATWRKY24	AT5G41570.1	regulation of transcription, DNA-templated, transcription, DNA-templated
C.cajan_29457	CcWRKY69	XP_020237140.1	ATWRKY69	AT3G58710.2	regulation of transcription, DNA-templated, transcription, DNA-templated
C.cajan_29780	CcWRKY7	KYP48318.1	ATWRKY7	AT4G24240.1	Encodes a Ca-dependent calmodulin binding protein. Sequence similarity to the WRKY transcription factor gene family.
C.cajan_29944	CcWRKY33	KYP47430.1	ATWRKY33	AT2G38470.1	camalexin biosynthetic process, cellular heat acclimation, cellular response to heat, defense response to bacterium, defense response to fungus, positive regulation of autophagy, regulation of transcription, DNA-templated, response to chitin, response to cold, response to heat, response to osmotic stress, response to salt stress, response to water deprivation, transcription, DNA-templated
C.cajan_30708	CcWRKY75	XP_020237079.1	ATWRKY75	AT5G13080.1	atrichoblast differentiation, lateral root development, negative regulation of transcription by RNA polymerase II, regulation of DNA-templated transcription in response to stress, regulation of response to nutrient levels, regulation of transcription, DNA-templated, transcription, DNA-templated
C.cajan_32192	CcWRKY40	XP_020237885.1	ATWRKY40	AT1G80840.1	defense response to bacterium, defense response to fungus, negative regulation of transcription, DNA-templated, regulation of defense response, regulation of defense response to virus by host, regulation of transcription, DNA-templated, response to chitin, response to molecule of bacterial origin, response to salicylic acid, response to wounding, transcription, DNA-templated
C.cajan_32591	CcWRKY70	XP_020237415.1	ATWRKY70	AT3G56400.1	defense response to bacterium, defense response to fungus, induced systemic resistance, jasmonic acid mediated signaling pathway, negative regulation of leaf senescence, negative regulation of transcription, DNA-templated, regulation of defense response, regulation of transcription, DNA-templated, response to chitin, response to jasmonic acid, response to salicylic acid,

					systemic acquired resistance, salicylic acid mediated signaling pathway, transcription, DNA-templated
C.cajan_32672	CcWRKY49	KYP44888.1	ATWRKY49	AT5G43290.1	regulation of transcription, DNA-templated, transcription, DNA-templated
C.cajan_35299	CcWRKY6	KYP43800.1	ATWRKY6	AT1G62300.1	cellular response to boron-containing substance deprivation, cellular response to phosphate starvation, ethylene-activated signaling pathway, negative regulation of transcription, DNA-templated, regulation of transcription, DNA-templated, response to chitin, transcription, DNA-templated.
C.cajan_35643	CcWRKY32	KYP41386.1	ATWRKY32	AT4G30935.1	regulation of transcription, DNA-templated, transcription, DNA-templated
C.cajan_36768	CcWRKY50	KYP37592.1	ATWRKY50	AT5G26170.1	defense response to fungus, jasmonic acid mediated signaling pathway, regulation of transcription, DNA-templated, transcription, DNA-templated
C.cajan_37436	CcWRKY47	XP_020202307.1	ATWRKY42	AT4G04450.1	negative regulation of transcription, DNA-templated, regulation of transcription, DNA-templated, transcription, DNA-templated
C.cajan_39263	CcWRKY12	KYP41601.1	ATWRKY12	AT2G44745.1	regulation of transcription, DNA-templated, transcription, DNA-templated
C.cajan_39557	CcWRKY20	KYP35093.1	ATWRKY20	AT4G26640.2	positive regulation of transcription, DNA-templated, regulation of transcription, DNA-templated, response to 1-aminocyclopropane-1-carboxylic acid, response to wounding, transcription, DNA-templated
C.cajan_40644	CcWRKY51	KYP34727.1	ATWRKY51	AT5G64810.1	defense response to bacterium, defense response to fungus, jasmonic acid mediated signaling pathway, regulation of transcription, DNA-templated, transcription, DNA-templated
C.cajan_40968	CcWRKY65	KYP38919.1	ATWRKY65	AT1G29280.1	regulation of transcription, DNA-templated, transcription, DNA-templated
C.cajan_41159	CcWRKY72	KYP39644.1	ATWRKY72	AT5G15130.1	defense response, regulation of transcription, DNA-templated, transcription, DNA-templated
C.cajan_41239	CcWRKY11	XP_020207515.1	ATWRKY11	AT4G31550.1	defense response to bacterium, regulation of transcription, DNA-templated, response to chitin, transcription, DNA-templated
C.cajan_41592	CcWRKY50	XP_020206759.1	ATWRKY50	AT5G26170.1	defense response to fungus, jasmonic acid mediated signaling pathway, regulation of transcription, DNA-templated, transcription, DNA-templated
C.cajan_42797	CcWRKY57	KYP36787.1	ATWRKY57	AT1G69310.2	regulation of transcription, DNA-templated, response to osmotic stress, response to salt stress, response to water deprivation, transcription, DNA-templated

C.cajan_43040	CcWRKY12	XP_020206980.1	ATWRKY12	AT2G44745.1	regulation of transcription, DNA-templated, transcription, DNA-templated
C.cajan_43923	CcWRKY75	KYP33931.1	ATWRKY75	AT5G13080.1	atrichoblast differentiation, lateral root development, negative regulation of transcription by RNA polymerase II, regulation of DNA-templated transcription in response to stress, regulation of response to nutrient levels, regulation of transcription, DNA-templated, transcription, DNA-templated
C.cajan_44344	CcWRKY40	XP_020206660.1	ATWRKY40	AT1G80840.1	defense response to bacterium, defense response to fungus, negative regulation of transcription, DNA-templated, regulation of defense response, regulation of defense response to virus by host, regulation of transcription, DNA-templated, response to chitin, response to molecule of bacterial origin, response to salicylic acid, response to wounding, transcription, DNA-templated
C.cajan_44345	CcWRKY40	KYP34952.1	ATWRKY18	AT4G31800.1	defense response to bacterium, defense response to fungus, regulation of defense response, regulation of defense response to virus by host, regulation of transcription, DNA-templated, response to chitin, response to molecule of bacterial origin, response to salicylic acid, transcription, DNA-templated
C.cajan_47800	CcWRKY70	XP_020209514.1	ATWRKY68	AT3G62340.1	regulation of transcription, DNA-templated, transcription, DNA-templated
C.cajan_48561	CcWRKY70	XP_020209657.1	ATWRKY70	AT3G56400.1	defense response to bacterium, defense response to fungus, induced systemic resistance, jasmonic acid mediated signaling pathway, negative regulation of leaf senescence, negative regulation of transcription, DNA-templated, regulation of defense response, regulation of transcription, DNA-templated, response to chitin, response to jasmonic acid, response to salicylic acid, systemic acquired resistance, salicylic acid mediated signaling pathway,

Figure SF1: Motif analysis detail of CcWRKYs

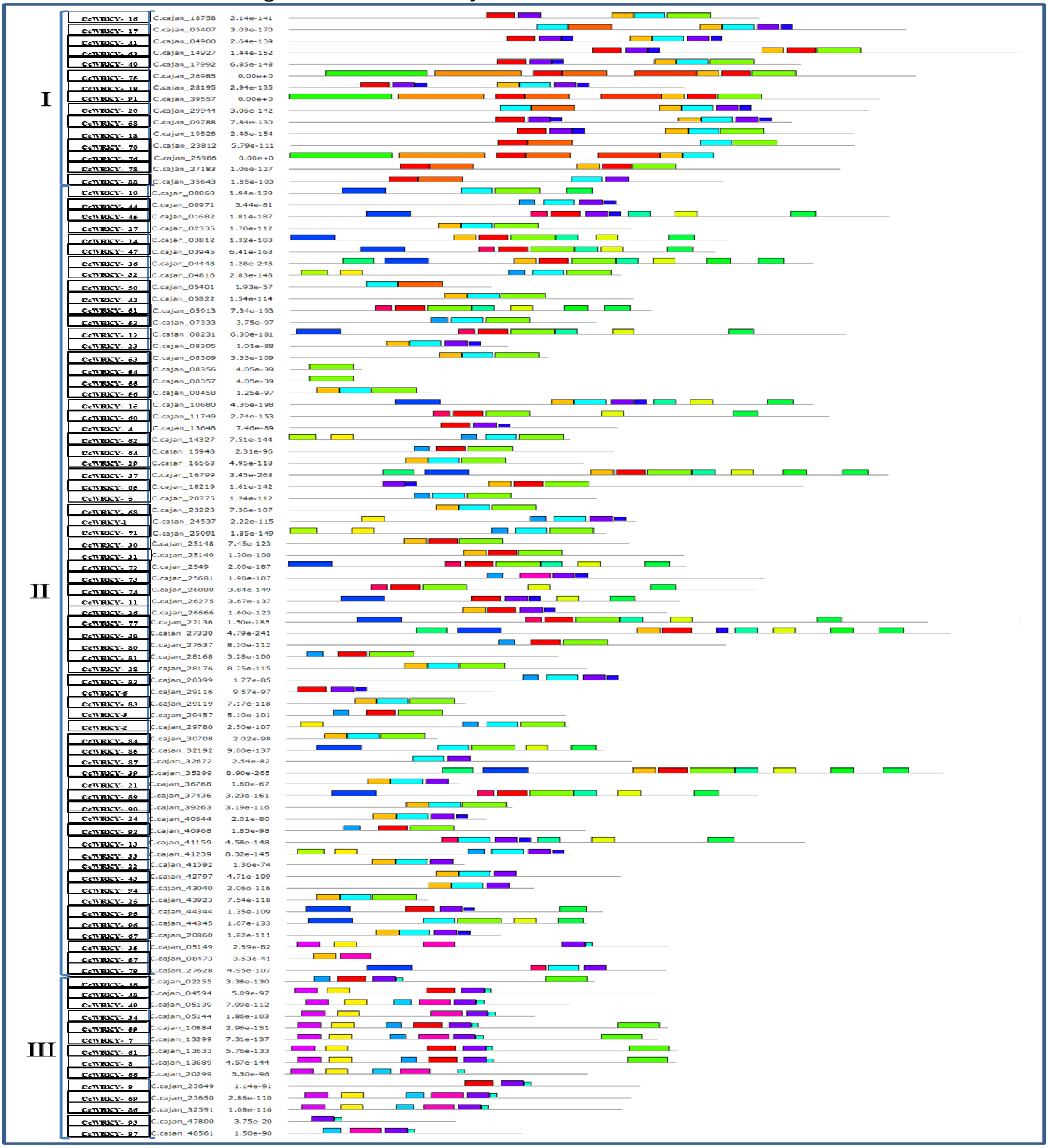

Motif1	DDGYQWRKYGQKQVKGNPYPRSYYRCT
Motif2	ILDDGYRWRKYGQKVVKGSPYPRSYYKCT
Motif3	PGCPVRKQVZRSSEDPSIVITTYEGTHNHPLPAAANSSAST
Motif4	CPVKKQVZRSLEDPTMVITTY
Motif5	KKGEKKVREPRVAFQTRSEVD
Motif6	AAITADPNFTAALAAAISSIIGGA
Motif7	TLQAELERVKEENKRLREMLNQVNEBYNALQMHLMEJMQQQ
Motif8	DGYAWRKYGQKEILNSKFPRSYYRCTHKY
Motif9	HPNCEVKKLVERSHDGQITEIVYKGTHNHPKPQPTRRYSSS
Motif10	MATLSASAPFPTITLDLTQPP
Motif11	MSPAKLPISRSPCVTIPPGLSPTSFLESPVLLSNMKVEPSPTTGSLSMLHQTAHGSVTSAASATFPVTTACFNSNTVDDRKPSFFEFKPHSRSNM
Motif12	TSAAASMLLSGSMTSSDGJMN
Motif13	KKRKNRVKRVVRVPA
Motif14	GEHNHPKPPAR
Motif15	JIEELVQGQEVATQLKGLLENP
Motif16	PIKKARVSVRARCDT
Motif17	QMSHPADPDSTPELSPVATNDGDLEGTGFLSNRNTDEVDDDDPFSKRRKMDLGNADI
Motif18	DNHASEKSTQIDGQGKTQAFDSSPFVKNEIADPSNELSLSSPVQMVSSGAGARVEGDLDELNPRSNIATGLQASQVDNRG
Motif19	LVDNVLSSFKKAJSJLNRTGT
Motif20	MEEQIAIQEAASEGLKGMEHLIRLL
Motif21	SDSDITDIISAPTSATNSPILDLDFSLDKVDFDTEFPF
Motif22	PQIFGQALYNQSKFSGLQLSQD
Motif23	VNTGLNLLTANTGSDQSTVDDGISPNAED
Motif24	RGRHTCT
Motif25	KNRRGCYKRRRTEQTWE

Table S3: Motifs with their function in CcWRKYs

Motifs No.	Sequences	Possible Match	Predicted function	References
Motif 1	DDGYQWRKYGQKQVKGNPYPRSYYRCT	Solution Structure Of The C-Terminal Wrky Domain Of Atwrky4	defense response, negative regulation of defense response to bacterium, regulation of defense response to fungus, regulation of transcription, DNA-templated, response to ethylene, response to jasmonic acid, response to salicylic acid, transcription, DNA-templated	[Yamasaki et al. 2005]
Motif 2	ILDDGYRWRKYGQKVVKGSPYPRSYYKCT	Solution Structure Of The C-Terminal Wrky Domain Of Atwrky4	defense response, negative regulation of defense response to bacterium, regulation of defense response to fungus, regulation of transcription, DNA-templated, response to ethylene, response to jasmonic acid, response to salicylic acid, transcription, DNA-templated	[Yamasaki et al. 2005]
Motif 3	PGCPVRKQVZRSSEDPSIVITTYEGTHNHPLPAAANSSAST	Solution Structure Of The C-Terminal Wrky Domain Of Atwrky4	defense response, negative regulation of defense response to bacterium, regulation of defense response to fungus, regulation of transcription, DNA-templated, response to ethylene, response to jasmonic acid, response to salicylic acid, transcription, DNA-templated	[Yamasaki et al. 2005]
Motif 4	CPVKKQVZRSLEDPTMVITTY	Crystal Structure Of The C-terminal Wrky Domain of Atwrky1, An Sa- Induced And Partially Npr1-dependent Transcription Factor	positive regulation of transcription, DNA-templated, regulation of transcription, DNA-templated, salicylic acid mediated signaling pathway, transcription, DNA-templated	[Duan et al. 2007]
Motif 5	KKGEKKVREPRVAFQTRSEVD	plastid-specific ribosomal proteins in spinach	structural and functional<br>roles that the PSRPs play during protein synthesis in chloroplasts	[Sharma et al. 2007]
Motif 6	AAITADPNFTAALAAAISSIIGGA	Flexible IgE epitope-containing domains of Phl p 5	cause high allergenic activity	[Göbl et al. 2017]
Motif 7	TLQAELERVKEENKRLREMLNQVNEBYNALQMHLMEJMQQQ			No similarity found
Motif 8	DGYAWRKYGQKEILNSKFPRSYYRCTHKY	Crystal Structure Of The C-terminal Wrky Domain of Atwrky1, An Sa- Induced And Partially Npr1-	positive regulation of transcription, DNA-templated, regulation of transcription, DNA-templated, salicylic acid mediated signaling pathway, transcription, DNA-templated / PopP2-	[Duan et al. 2007 and Zhang et al. 2017]

		dependent Transcription Factor / Chain B, Crystal structure of PopP2 in complex with IP6, AcCoA and the WRKY domain of RRS1-R	mediated acetylation, an acetyl-cysteine covalent adduct, lending direct support to the 'ping-pong'-like catalytic mechanism proposed for YopJ effectors.	
Motif 9	HPNCEVKKLVERS HDGQITEIVYKGTH NHPKPQPTRRYSSS	-	-	
Motif 10	MATLSASAPFPTITL DLTQPP	Chain A, Crystal Structure Of Glutathione Transferase Lambda3 From *Populus Trichocarpa*	play crucial roles in detoxification processes and secondary metabolism	[Lallement et al. 2010]
Motif 11	MSPAKLPISRSPCV TIPPGLSPTSFLESP VLLSNMKVEPSPTT GSLSMLHQTAHGS VTSAASATFPVTTA CFNSNTVDDRKPSF FEFKPHSRSNM	Chain A, Crystal Structure Of A 6-Sst6-Sft From *Pachysandra Terminalis*	Stress protectants in plants, and additionally serve as prebiotics with emerging antioxidant properties.	[Lammens et al. 2012]
Motif 12	TSAAASMLLSGSM TSSDGJMN	Chain B, Crystal structure of potassium-dependent plant-type L-asparaginase from *Phaseolus vulgaris* in complex with K+ cations	The enzymes are heat- stable up to 60 °C and show both isoaspartyl di-peptidase andL-asparaginase activities.	[Borek et al. 2004]
Motif 13	KKRKNRVKRVVRV PA	Chain B, Crystal structure of PopP2 in complex with IP6, AcCoA and the WRKY domain of RRS1-R	PopP2 recognizes the WRKYGQK motif of RRS1-R_{WRKY} to position a targeted lysine in the active site for acetylation.	[Zhang et al. 2017]
Motif 14	GEHNHPKPPAR	Chain B, Crystal structure of PopP2 in complex with IP6, AcCoA and the WRKY domain of RRS1-R	PopP2 recognizes the WRKYGQK motif of RRS1-R_{WRKY} to position a targeted lysine in the active site for acetylation.	[Zhang et al. 2017]
Motif 15	JIEELVQGQEVATQ LKGLLENP	Chain A, Crystal Structure of nuclear proteinaceous RNase P 2 (PRORP2) from *A. thaliana*	RNA processing enzymes that catalyze maturation of the 5' end of precursor tRNAs in Eukaryotes.	[Karasik et al. 2016]
Motif 16	PIKKARVSVRARCD T	plastid-specific ribosomal proteins	structural and functional roles that the PSRPs play during protein synthesis in chloroplasts	[Sharma et al. 2007]
Motif 17	QMSHPADPDSTPEL SPVATNDG	-	-	-

	DLEGTGFLSNRNTD EVDDDDPFSKRRK MDLGNADI			
Motif 18	DNHASEKSTQIDGQ GKTQAFDSSPFVKN EIADPSN ELSLSSPVQMVSSG AGARVEGDLDELN PRSNIATGLQASQV DNRG	-	-	-
Motif 19	LVDNVLSSFKKAJS JLNRTGT	Terpene synthases	Responsible for the biosynthesis of the complex chemical defense arsenal of plants and microorganisms	[Kampranis et al. 2007]
Motif 20	MEEQIAIQEAASEG LKGMEHLIRLL	Ferredoxin NADP(+) reductase	This flavoprotein is the last enzyme in the electron transport chain of linear photosynthesis where electrons are transferred through a series of electron carriers and finally produce NADPH	[Dorowski et al. 2001]
Motif 21	SDSDITDIISAPTSAT NSPILDLDFSLDKV DFDTEFPF	-	-	-
Motif 22	PQIFGQALYNQSKF SGLQLSQD	Ribonuclease P (RNase P)	Typically these enzymes are ribonucleoproteins with a conserved RNA component responsible for catalysis.	[Howard et al. 2012]
Motif 23	VNTGLNLLTANTG SDQSTVDDGISPNA ED	cardosin B	this enzyme may be involved in the remodeling and/or degradation of the pistil extracellular matrix during pollen tube growth	[Vieira et al. 2001]
Motif 24	RGRHTCT	TOPLESS (TPL) and TOPLESS-related (TPR) proteins	In plants, TPL/TPR corepressors regulate development, stress responses, and hormone signaling through interaction with small ethylene response factor-associated amphiphilic repression (EAR) motifs found in diverse transcriptional repressors	[Ke et al. 2015]
Motif 25	KNRRGCYKRRRTE QTWE	*Arabidopsis thaliana* (5-Methylthioribose (MTR)	5-Methylthioribose (MTR) kinase is a key enzyme required for methionine salvage in plants and many bacteria	[Ku et al. 2007]

References

Yamasaki, K., Kigawa, T., Inoue, M., Tateno, M. Yamasaki, T., Yabuki, T. and Hayami, N. (2005) Solution structure of an *Arabidopsis* WRKY DNA binding domain. *The Plant Cell* 17: 944-956.

Duan, M.R., Nan, J., Liang, Y.H., Mao, P., Lu, L., Li, L. and Su, X.D. (2007) DNA binding mechanism revealed by high resolution crystal structure of *Arabidopsis thaliana* WRKY1 protein. *Nucleic Acids Res.* 35: 1145-1154.

Sharma, M.R., Wilson, D.N., Datta, P.P., Barat, C., Schluenzen, F., Fucini, P. and Agrawal, R.K. (2007) Cryo-EM study of the spinach chloroplast ribosome reveals the structural and functional roles of plastid-specific ribosomal proteins. *P. Natl. A. Sci.* 104: 19315-19320.

Göbl, C., Focke-Tejkl, M., Najafi, N., Schrank, E., Madl, T., Kosol, S. and Valenta, R. (2017) Flexible IgE epitope-containing domains of Phl p 5 cause high allergenic activity. *J. Allergy Clin. Immun.* 140: 1187-1191.

Zhang, Z.M., Ma, K.W., Gao, L., Hu, Z., Schwizer, S., Ma, W. and Song, J. (2017) Mechanism of host substrate acetylation by a YopJ family effector. *Nat. plants* 3: 17115.

Lallement, P.A., Meux, E., Gualberto, J.M., Prosper, P., Didierjean, C., Saul, F. and Hecker, A. (2014) Structural and enzymatic insights into Lambda glutathione transferases from *Populus trichocarpa*, monomeric enzymes constituting an early divergent class specific to terrestrial plants. *Biochem. J.* 462: 39-52.

Lammens, W., Le Roy, K., Yuan, S., Vergauwen, R., Rabijns, A., Van Laere, A. and Van den Ende, W. (2012) Crystal structure of 6-SST/6-SFT from *Pachysandra terminalis*, a plant fructan biosynthesizing enzyme in complex with its acceptor substrate 6-kestose. *The Plant J.* 70: 205-219.

Borek, D., Michalska, K., Brzezinski, K., Kisiel, A., Podkowinski, J., Bonthron, D.T. and Jaskolski, M. (2004) Expression, purification and catalytic activity of *Lupinus luteus* asparagine β-amidohydrolase and its *Escherichia coli* homolog. *The FEBS J.* 271: 3215-3226.

Karasik, A., Shanmuganathan, A., Howard, M.J., Fierke, C.A. and Koutmos, M. (2016) Nuclear protein-only Ribonuclease P2 structure and biochemical characterization provide insight into the conserved properties of tRNA 5′ end processing enzymes. *J. Mol. Biol.* 428: 26-40.

Sharma, M.R., Wilson, D.N., Datta, P.P., Barat, C., Schluenzen, F. Fucini, P. and Agrawal, R.K. (2007) Cryo-EM study of the spinach chloroplast ribosome reveals the structural and functional roles of plastid-specific ribosomal proteins. *P. Natl. A. Sci.* 104: 19315-19320.

Kampranis, S.C., Ioannidis, D., Purvis, A., Mahrez, W., Ninga, E., Katerelos, N.A. and Goodenough, P.W. (2007) Rational conversion of substrate and product specificity in a Salvia monoterpene synthase: structural insights into the evolution of terpene synthase function. *The Plant Cell* 19: 1994-2005.

Dorowski, A., Hofmann, A., Steegborn, C., Boicu, M. and Huber, R. (2001) Crystal structure of paprika ferredoxin-NADP+ reductase Implications for the electron transfer pathway. *J. Biol. Chem.* 276: 9253-9263.

Howard, M.J., Lim, W.H., Fierke, C.A. and Koutmos, M. (2012) Mitochondrial ribonuclease P structure provides insight into the evolution of catalytic strategies for precursor-tRNA 5′ processing. *P. Natl. A. Sci.* 109: 16149-16154.

Vieira, M., Pissarra, J., Verissimo, P., Castanheira, P., Costa, Y., Pires, E.and Faro, C. (2001) Molecular cloning and characterization of cDNA encoding cardosin B, an aspartic proteinase accumulating extracellularly in the transmitting tissue of *Cynara cardunculus* L. *Plant Mol. Biol.* 45: 529–539.

Ke, J., Ma, H., Gu, X., Thelen, A., Brunzelle, J.S., Li, J. and Melcher, K. (2015) Structural basis for recognition of diverse transcriptional repressors by the TOPLESS family of corepressors. *Science Advances* 1: e1500107.

Ku, S.Y., Cornell, K.A. and Howell, P.L. (2007) Structure of Arabidopsis thaliana 5-methylthioribose kinase reveals a more occluded active site than its bacterial homolog. *BMC Struct. Biol.* 7: 70.

Figure SF2: CcWRKYs domain and protein analysis

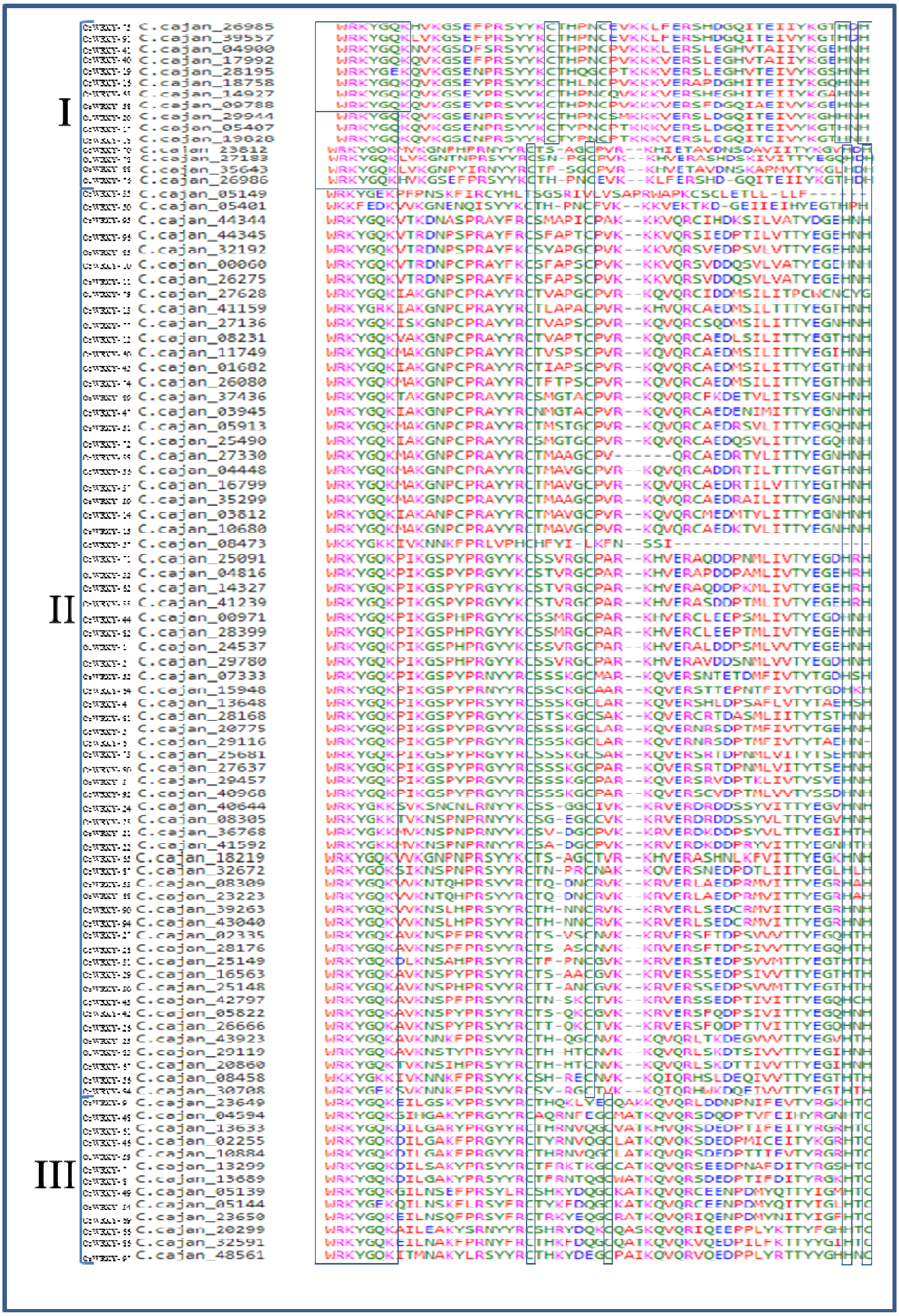

Table S4: CcWRKYs physicochemical analysis

S. No.	Accession no.	AAR	MW	PI	TA	EC	II	AI	GRAVY
1.	C.cajan_00060	278	31165.06	8.57	4336	23295	50.50	64.46	-0.772
2.	C.cajan_00971	302	33949.38	9.68	4715	16555	58.98	64.87	-0.804
3.	C.cajan_01682	603	65777.32	6.17	9102	35590	50.54	60.40	-0.695
4.	C.cajan_02255	643	70224.20	7.25	9687	52800	55.37	59.83	-0.522
5.	C.cajan_02335	313	35301.07	6.27	4858	23630	57.71	55.08	-0.861
6.	C.cajan_03812	400	44226.72	9.27	6177	27890	49.42	73.95	-0.294
7.	C.cajan_03945	389	43186.79	7.94	6015	28475	60.41	76.04	-0.489
8.	C.cajan_04448	478	51578.33	6.50	7138	16305	42.62	68.24	-0.603
9.	C.cajan_04594	344	38516.08	6.41	5319	31650	55.64	66.34	-0.576
10.	C.cajan_04816	304	32961.73	9.64	4639	14690	43.85	66.04	-0.545
11.	C.cajan_04900	452	49718.55	6.83	6884	37735	45.73	63.63	-0.668
12.	C.cajan_05139	263	29741.10	6.83	4100	39795	48.67	60.80	-0.860
13.	C.cajan_05144	231	26096.32	6.43	3659	30160	46.19	81.90	-0.361
14.	C.cajan_05149	348	39214.58	5.53	5389	51505	45.75	66.09	-0.672
15.	C.cajan_05401	185	21369.70	6.29	2941	22140	42.34	65.84	-0.848
16.	C.cajan_05407	571	62356.32	6.48	8543	53205	64.76	47.67	-0.877
17.	C.cajan_05822	586	65482.62	6.22	8933	48415	65.59	46.95	-1.042
18.	C.cajan_05913	617	66563.98	5.75	9158	33640	53.63	61.10	-0.655
19.	C.cajan_07333	652	71549.55	5.68	9804	55975	59.64	51.06	-0.874
20.	C.cajan_08231	509	55435.06	8.36	7704	28140	44.77	66.90	-0.551
21.	C.cajan_08305	199	22617.64	6.13	3062	34630	56.07	42.56	-1.078
22.	C.cajan_08309	236	26807.67	8.79	3707	29450	51.55	59.45	-0.742
23.	C.cajan_08356	67	8122.45	7.77	1104	21095	66.99	49.40	-0.907
24.	C.cajan_08357	66	7935.92	7.77	1080	15595	67.86	50.15	-0.908
25.	C.cajan_08458	133	15347.11	8.42	2124	16055	47.80	65.86	-0.741
26.	C.cajan_08473	84	9884.19	9.52	1379	12950	52.41	64.88	-0.677
27.	C.cajan_09788	466	51511.05	8.85	7093	37860	47.03	52.73	-0.914
28.	C.cajan_10680	478	52303.75	8.59	7258	25035	41.82	68.49	-0.515
29.	C.cajan_10884	354	40497.55	5.75	5614	28795	56.02	65.00	-0.666
30.	C.cajan_11749	493	53784.75	5.92	7412	41995	57.69	64.16	-0.587
31.	C.cajan_13299	344	38313.90	6.12	5302	30535	49.24	67.15	-0.593
32.	C.cajan_13633	363	40342.75	5.56	5562	30035	66.95	62.62	-0.675
33.	C.cajan_13648	300	33712.87	5.88	4685	39795	39.89	62.73	-0.676
34.	C.cajan_13689	361	40889.41	5.63	5624	45755	49.87	60.25	-0.758
35.	C.cajan_14327	256	28174.21	9.96	3971	14815	40.96	70.55	-0.619
36.	C.cajan_14927	678	73688.00	5.87	10082	47580	53.15	55.25	-0.840
37.	C.cajan_15948	296	32190.45	8.50	4431	20315	52.63	51.15	-0.852
38.	C.cajan_16563	269	29968.15	6.49	4127	23170	59.53	54.42	-0.898
39.	C.cajan_16799	547	58897.66	6.81	8162	25815	45.54	64.08	-0.646
40.	C.cajan_17992	474	51694.16	6.72	7110	38765	44.70	55.59	-0.833
41.	C.cajan_18219	469	51362.19	6.31	7066	29840	43.62	57.21	-0.740
42.	C.cajan_18758	436	48066.53	7.00	6656	29130	61.01	59.29	-0.828
43.	C.cajan_19828	523	57924.54	6.63	7934	58580	55.44	47.88	-0.939
44.	C.cajan_20299	279	32429.43	6.86	4462	34755	54.37	60.43	-0.936
45.	C.cajan_20775	281	31041.30	7.75	4276	25690	45.61	46.55	-0.867
46.	C.cajan_20860	195	22018.82	8.56	3047	18700	43.42	59.03	-0.792
47.	C.cajan_23223	234	26551.87	9.10	3671	24200	54.95	56.67	-0.670
48.	C.cajan_23649	322	35792.89	5.86	4923	26025	60.32	59.13	-0.743
49.	C.cajan_23650	313	35582.20	5.65	4892	31650	50.57	62.94	-0.810
50.	C.cajan_23812	515	55943.83	8.71	7804	32025	52.85	65.67	-0.756

51.	C.cajan_24537	316	34749.45	9.68	4872	19285	46.05	66.08	-0.660
52.	C.cajan_25091	289	31698.83	10.09	4407	14815	65.08	57.44	-0.671
53.	C.cajan_25148	316	34399.93	6.27	4747	24535	55.01	58.07	-0.812
54.	C.cajan_25149	367	40619.21	5.87	5610	24660	62.15	58.17	-0.778
55.	C.cajan_25490	369	40685.23	9.46	5699	27180	35.22	71.22	-0.560
56.	C.cajan_25681	442	48609.27	5.61	6645	41285	52.18	51.70	-0.859
57.	C.cajan_26080	434	47718.53	9.31	6630	39935	53.71	66.11	-0.544
58.	C.cajan_26275	363	40316.57	8.67	5637	28795	43.67	70.88	-0.582
59.	C.cajan_26666	351	38491.30	7.29	5290	24995	55.54	52.79	-0.837
60.	C.cajan_26985	580	62857.27	6.32	8613	33850	44.93	52.31	-0.822
61.	C.cajan_26986	451	49603.87	7.26	6821	8890	48.93	55.57	-0.845
62.	C.cajan_27136	594	64825.50	6.23	8935	34880	54.76	57.53	-0.863
63.	C.cajan_27183	510	56077.80	6.00	7749	26775	48.95	63.59	-0.923
64	C.cajan_27330	606	65713.48	5.83	9005	23295	48.52	59.64	-0.740
65	C.cajan_27628	346	39614.59	5.53	5492	27555	60.22	64.25	-0.880
66	C.cajan_27637	401	43916.38	5.22	6029	37400	50.06	62.52	-0.714
67	C.cajan_28168	248	28445.62	5.16	3932	21805	62.13	54.64	-1.044
68	C.cajan_28176	274	30528.92	7.69	4202	23630	64.46	54.05	-0.779
69	C.cajan_28195	365	40489.16	9.13	5567	35090	43.30	47.53	-0.933
70	C.cajan_28399	307	34204.75	9.77	4766	13575	43.08	66.35	-0.745
71	C.cajan_29116	189	20704.68	4.74	2832	24200	46.86	51.11	-0.832
72	C.cajan_29119	163	18755.27	9.32	2618	20190	33.29	62.15	-0.803
73	C.cajan_29457	255	27545.69	6.01	3812	28670	58.11	58.98	-0.669
74	C.cajan_29780	257	28904.90	9.91	4060	16305	55.01	63.35	-0.804
75	C.cajan_29944	536	59680.20	7.20	8140	60195	57.80	44.01	-0.968
76	C.cajan_30708	137	16184.36	9.58	2267	28545	59.48	68.91	-0.877
77	C.cajan_32192	289	32120.93	7.04	4471	19285	49.05	71.80	-0.609
78	C.cajan_32591	305	34928.57	5.44	4794	31900	70.43	57.80	-0.832
79	C.cajan_32672	315	35082.96	7.11	4891	49765	39.48	77.43	-0.399
80	C.cajan_35299	599	64850.05	6.04	8948	20315	49.48	62.64	-0.671
81	C.cajan_35643	396	43206.35	8.87	6020	28015	54.89	62.25	-0.794
82	C.cajan_36768	159	18412.16	5.24	2511	23045	28.05	45.22	-1.083
83	C.cajan_37436	431	47337.47	7.61	6609	18045	48.61	75.66	0.528
84	C.cajan_39263	207	23386.69	8.86	3196	29825	48.88	50.72	-0.904
85	C.cajan_39557	547	59098.58	7.32	8157	33850	46.59	59.71	-0.675
86	C.cajan_40644	183	20843.81	8.25	2832	25120	49.43	41.58	-1.011
87	C.cajan_40968	273	30611.03	5.03	4212	38305	66.16	52.12	-0.908
88	C.cajan_41159	474	52340.95	7.91	7256	37650	51.89	63.86	-0.597
89	C.cajan_41239	262	28999.07	9.56	4077	14690	46.73	68.93	-0.626
90	C.cajan_41592	161	18629.15	5.54	2520	27055	35.86	36.83	-1.338
91	C.cajan_42797	305	33556.07	6.46	4601	34295	62.55	50.00	-0.818
92	C.cajan_43040	226	25526.04	6.70	3477	32805	51.60	52.17	-0.897
93	C.cajan_43923	129	14756.74	9.81	2071	16055	29.25	62.56	-0.881
94	C.cajan_44344	288	32462.91	8.19	4571	18700	47.60	81.87	-0.642
95	C.cajan_44345	273	30754.76	8.76	4314	20315	56.63	77.47	-0.565
96	C.cajan_47800	153	17708.64	4.86	2412	16640	62.00	64.25	-0.527
97	C.cajan_48561	214	24731.62	5.70	3383	35215	55.54	55.98	-0.739

Abbreviation:- AAR-Amino acid residue, MW- Molecular weight, PI- Isoelectric Point, TA- Total number of atoms, EC- Extinction coefficients, II- Instability index, AI- Aliphatic index, A- Aromaticity, GRAVY- Grand average of hydropathicity.

Table S5: Expression profile of pathogen responsive *CcWRKY* genes in pigeonpea cultivars Asha and Bahar under different treatments

Treatments	Gene regulation	CcWRKY genes in pigeonpea cultivars
**P**	**Up-regulated**	**Asha:** *CcWRKY*s 1, 3, 4, 5, 7, 10, 11, 12, 13, 14, 15, 20, 21, 22, 23, 24, 25, 26, 27, 28, 29, 30, 31, 32, 33, 34, 35, 36, 37, 38, 39, 40, 41, 42, 43
		**Bahar:** *CcWRKY*s 1, 3, 4, 5, 7, 10, 11, 12, 13, 14, 15, 21, 23, 24, 25, 26, 27, 28, 29, 30, 31, 32, 35, 38, 40, 41
	**Down regulated**	**Asha:** None
		**Bahar:** *CcWRKY*s 20, 22, 33, 34, 36, 37, 39, 42, 43
**S**	**Up-regulated**	**Asha:** *CcWRKY*s 1, 3, 4, 5, 7, 10, 11, 12, 13, 14, 15, 20, 21, 22, 23, 24, 26, 27, 28, 29, 30, 31, 32, 33, 34, 35, 36, 37, 38, 39, 40, 41, 42, 43
		**Bahar:** *CcWRKY*s 1, 3, 4, 5, 7, 10, 11, 12, 13, 14, 15, 20, 23, 24, 25, 26, 27, 28, 29, 38
	**Down regulated**	**Asha:** *CcWRKY*s25
		**Bahar:** *CcWRKY*s 21, 22, 30, 31, 32, 33, 34, 35, 36, 37, 39, 40, 41, 42, 43
**O**	**Up-regulated**	**Asha:** *CcWRKY*s 1, 3, 4, 5, 7, 10, 11, 12, 13, 14, 15, 20, 21, 22, 23, 24, 25, 26, 27, 28, 29, 30, 31, 32, 33, 34, 35, 36, 37, 38, 39, 40, 41, 42, 43
		**Bahar:** *CcWRKY*s 1, 3, 4, 5, 7, 10, 11, 12, 13, 14, 15, 20, 21, 23, 24, 25, 26, 27, 28, 29, 30, 31, 32, 33, 34, 35, 36, 37, 39, 40, 41, 42, 43
	**Down regulated**	**Asha:** None
		**Bahar:** *CcWRKY*s 22, 38
**PS**	**Up-regulated**	**Asha:** *CcWRKY*s 1, 3, 4, 5, 7, 10, 11, 12, 13, 14, 15, 20, 21, 22, 23, 24, 26, 27, 28, 29, 30, 31, 32, 33, 34, 35, 36, 37, 38, 39, 40, 41, 42, 43
		**Bahar:** *CcWRKY*s 1, 3, 4, 5, 7, 10, 11, 12, 13, 14, 15, 20, 23, 24, 25, 26, 27, 28, 29, 30, 35, 38, 40, 41, 43
	**Down regulated**	**Asha:** *CcWRKY* 25
		**Bahar:** *CcWRKY*s 21, 22, 31, 32, 33, 34, 36, 37, 39, 42
**PO**	**Up-regulated**	**Asha:** *CcWRKY*s 1, 3, 4, 5, 7, 10, 11, 12, 13, 14, 15, 20, 21, 22, 23, 24, 26, 27, 28, 29, 30, 31, 32, 33, 34, 35, 36, 37, 38, 39, 40, 41, 42, 43
		**Bahar:** *CcWRKY*s 1, 3, 4, 5, 7, 10, 11, 12, 13, 14, 15, 20, 23, 24, 25, 26, 27, 28, 29, 35, 38, 41, 43
	**Down regulated**	**Asha:** *CcWRKY*25
		**Bahar:** *CcWRKY*s 21, 22, 30, 31, 32, 33, 34, 36, 37, 39, 40, 42
**SO**	**Up-regulated**	**Asha:** CcWRKY-1,3,4,5,7,10,11,12,13,14,15,20,21,22,23,24,25,26,27,28,29,30, 31,32,33,34,35,36,37,38,39,40,41,42,43
		**Bahar:** *CcWRKY*s 1, 3, 4, 5, 7, 10, 11, 12, 13, 14, 15, 20, 23, 24, 25, 26, 27, 28, 29, 30, 35, 36, 43
	**Down regulated**	**Asha:** None
		**Bahar:** *CcWRKY*s 21, 22, 31, 32, 33, 34, 37, 38, 39, 40, 41, 42
**PSO**	**Up-regulated**	**Asha:** *CcWRKY*s 1, 3, 4, 5, 7, 10, 11, 12, 13, 14, 15, 20, 21, 22, 23, 24, 25, 26, 27, 28, 29, 30, 31, 32, 33, 34, 35, 36, 37, 38, 39, 41, 42, 43
		**Bahar:** *CcWRKY*s 1, 3, 4, 5, 7, 10, 11, 12, 13, 14, 15, 20, 23, 24, 25, 26, 27, 28, 29
	**Down regulated**	**Asha:** *CcWRKY* 40
		**Bahar:** *CcWRKY*s 21, 22, 30, 31, 32, 33, 34, 35, 36, 37, 38, 39, 40, 41, 42, 43

Table S6: Fold change of pathogen responsive *CcWRKY* genes in pigeonpea cultivars Asha (A) and Bahar (B) under different treatments

Treatments	Cc WRKY genes fold change										
	**< 1**	**1-5**	**5-10**	**10-15**	**15-20**	**20-25**	**25-30**	**30-35**	**35-40**	**40-45**	**45-50**	**>50**
**AP**		*CcWRKY*s 3, 5, 11, 14, 15, 20, 21, 23, 24, 25, 26, 27, 29, 34, 35, 37, 38, 40, 42	*CcWRKY*s 10, 13, 28, 31, 33, 36, 41	*CcWRKY*s 1, 12, 32, 43	*CcWRKY*s 7, 22	*CcWRKY*30						*CcWRKY*s 4, 39
**BP**	*CcWRKY*s 20, 22, 33, 34, 36, 37, 39, 42, 43	*CcWRKY*s 1, 3, 4, 5, 7, 10, 14, 15, 21, 23, 24, 25, 26, 27, 28, 29, 30, 32, 35, 38, 40	*CcWRKY*s 12, 13	*CcWRKY*s 11, 31			*CcWRKY*s 41					
**AS**	*CcWRKY*s 25	*CcWRKY*s 5, 14, 24, 27, 33, 42	*CcWRKY*s 1, 3, 11, 13, 15, 20, 26, 35, 36, 40, 41	*CcWRKY*s 22, 28, 31, 32, 34, 38, 43	*CcWRKY*s 30, 37	*CcWRKY*s 10, 12, 29,	*CcWRKY*21	*CcWRKY*39		*CcWRKY*23		*CcWRKY*s 4, 7
**BS**	*CcWRKY*s 21, 22, 30, 31, 32, 33, 34, 35, 36, 37, 39, 40, 41, 42, 43	*CcWRKY*s 1, 3, 4, 5, 7, 15, 20, 23, 25, 26, 27, 28, 29	*CcWRKY*s 10, 12, 24	*CcWRKY*s 11, 13			*CcWRKY*s 14, 38					
**AO**		*CcWRKY*s 7, 14, 20, 25, 27, 28, 32, 41, 42	*CcWRKY*s 1, 4, 5, 13, 23, 30, 36, 38, 40,43	*CcWRKY*s 15, 24, 29,	*CcWRKY*s 22, 33	*CcWRKY*3	*CcWRKY*s 35, 39		*CcWRKY*s 26, 34		*CcWRKY*s 21	*CcWRKY*s 10, 11, 12, 31, 37
**BO**	*CcWRKY*s 22, 38	*CcWRKY*s 1, 3, 4, 5, 7, 10, 27, 29,30, 32, 35, 42	*CcWRKY*s 12, 20, 23, 28, 34, 36	*CcWRKY*s 15, 21, 33, 37, 40, 41	*CcWRKY*13	*CcWRKY*s 11, 14	*CcWRKY*s 25, 39		*CcWRKY*31			*CcWRKY*s 24, 26, 43
**APS**	*CcWRKY*25	*CcWRKY*s 1, 3, 4, 5, 7, 14, 15, 22, 23, 24, 27, 28, 29, 30, 32, 35, 38, 39, 41, 42, 43	*CcWRKY*s 20, 26, 31, 33, 36	*CcWRKY*s 13, 37	*CcWRKY*s 10, 11, 21	*CcWRKY* 40	*CcWRKY*s 34				*CcWRKY*12	
**BPS**	*CcWRKY*s 21, 22, 31, 32, 33, 34,	*CcWRKY*s 3, 4, 5, 7, 15, 20, 23, 24, 25, 27, 28,	*CcWRKY*s 10, 12, 26, 30	*CcWRKY*s 1, 11		*CcWRKY*13					*CcWRKY*14	*CcWRKY*38

	36, 37, 39, 42	29,35, 40, 41, 43										
**APO**	*CcWRKY*25	*CcWRKY*s 1, 3, 4, 5, 14, 24, 42, 43	*CcWRKY*s 7, 13, 15, 22, 26, 27, 37, 38, 40	*CcWRKY*s 21, 32, 36, 41	*CcWRKY*s 11, 23, 29,	*CcWRKY*s 20, 28, 31	*CcWRKY*s 10, 33, 35	*CcWRKY*34	*CcWRKY*30	*CcWRKY*12		*CcWRKY*39
**BPO**	*CcWRKY*s 21, 22, 30, 31, 32, 33, 34, 36, 37, 39, 40, 42	*CcWRKY*s 3, 4, 5, 10, 15, 20, 23, 24, 25, 26, 27, 28, 29, 35, 41, 43	*CcWRKY*s 7, 11, 12, 14	*CcWRKY*13		*CcWRKY*1						*CcWRKY*14
**ASO**		*CcWRKY*s 3, 5, 12, 14, 15, 24, 38, 42	*CcWRKY*s 4, 39, 40	*CcWRKY*s 1, 10, 23, 27, 33, 36	*CcWRKY*s 11, 26, 28, 31, 32, 41, 43	*CcWRKY*s 29, 35	*CcWRKY*s 13, 22	*CcWRKY*s 7, 37	*CcWRKY*20	*CcWRKY*30	*CcWRKY*s 21, 25, 34	
**BSO**	*CcWRKY*s 21, 22, 31, 32, 33, 34, 37, 38, 39, 40, 41, 42	*CcWRKY*s 4, 5, 7, 10,11, 13, 15, 20, 23, 25, 27, 28, 30, 35, 36, 43	*CcWRKY*s 3, 12, 24, 29	*CcWRKY*s 1, 26			*CcWRKY*14					
**APSO**	*CcWRKY*40	*CcWRKY*s 1, 3, 4, 5, 21, 24, 25, 26, 27, 33, 35, 37, 38, 41, 42, 43	*CcWRKY*s 11, 14, 15, 20, 22, 28, 30, 31, 32, 36	*CcWRKY*s 23, 39	*CcWRKY*s 13, 34	*CcWRKY*s 10, 29	*CcWRKY*s 7, 12					
**BPSO**	*CcWRKY*s 21, 22, 30, 31, 32, 33, 34, 35, 36, 37, 38, 39, 40, 41, 42, 43	*CcWRKY*s 5, 12, 15, 20, 23, 24, 26, 27, 28, 29	*CcWRKY*s 1, 3, 4, 10, 13, 25	*CcWRKY*s 7, 11			*CcWRKY14*					

Table S7: Primer details of CcWRKYs used in this study

Gene Accession no.	S.No.		Sequence	length	Product Size (bp)
C.cajan_24537	*CcWRKY1*	F	CTCTTTCTTCCTCTTCGTTG	20	130
		R	CTCACCACTCTCTTCAACCT	20	
C.cajan_29457	*CcWRKY3*	F	GAGATGAAGAAGAGGGTGGT	20	169
		R	CCTTTGAACTGCTGCATC	18	
C.cajan_13648	*CcWRKY4*	F	ATGGACAGAAACCGATAAAG	20	108
		R	GATCTAAGTGGCTCCTTTCA	20	
C.cajan_20775	*CcWRKY5*	F	ACTACCACCTCCTCCTCTTT	20	156
		R	AGAAGAGGTTTGGAAGGAGT	20	
C.cajan_13299	*CcWRKY7*	F	ATTATCCGTCCAAACAAGTC	20	198
		R	CTTCTGGGATATTTTGCACT	20	
C.cajan_00060	*CcWRKY10*	F	GAAGGTGCAAAGAAGTGTGGA	21	178
		R	AGTAGCCATCTGTTCCACCA	20	
C.cajan_26275	*CcWRKY11*	F	AGCACAATCATCCCCACCCTTCT	23	158
		R	AGACTCGCTTTTGGTGCTTGACT	23	
C.cajan_08231	*CcWRKY12*	F	CAACCATCCTCTTCCAGT	18	161
		R	GACTGAAACTCGGACCAT	18	
C.cajan_41159	*CcWRKY13*	F	ACCAACGTCCAAGAGCAAACCA	22	151
		R	TGATGGGATTCTTGGGGTTGAAGC	24	
C.cajan_03812	*CcWRKY14*	F	AACCACAACCACCCTCTT	18	196
		R	TAGTGGGAAATGGTGCAG	18	
C.cajan_10680	*CcWRKY15*	F	CGTCTCAATCATCACACC	18	159
		R	GTCCAGCACAGTTCTCAA	18	
C.cajan_29944	*CcWRKY20*	F	GTAATTCAGTTCCGAGGTCT	20	140
		R	ACTACGAGGATTTTCACTCC	20	
C.cajan_36768	*CcWRKY21*	F	TCGAGGGGTTTTCTAGCAGA	20	167
		R	ATAGTTCCTCGGATTGGGGC	20	

C.cajan_41592	*CcWRKY22*	F	CCACCACCAGATACACCT	18	186
		R	GCTGCTACCTCCAAAGTC	18	
C.cajan_08305	*CcWRKY23*	F	TGCAACCTCCACCATCAATAACA	23	174
		R	CTTGGGTTGGGGCTGTTCTT	20	
C.cajan_40644	*CcWRKY24*	F	TCACAAAGCACCACCGAGTC	20	192
		R	CTTGCCATACTTCCTCCACTTGT	23	
C.cajan_43923	*CcWRKY25*	F	CAGTGGTAGTGTTAGTGGTAGCA	23	164
		R	GGCCATACTTCCTCCATCGATAA	23	
C.cajan_26666	*CcWRKY26*	F	AGTGAAACCCTTGCGACCCT	20	181
		R	GCTCCTTCTGCTTCTTCTCTCCT	23	
C.cajan_02335	*CcWRKY27*	F	ACTGATCCAAGCGTTGTGGTGA	22	164
		R	TGATGGTTGTGGTGCTGGTGT	21	
C.cajan_28176	*CcWRKY28*	F	CAGTTATGGGTCGCTCGGGAG	21	184
		R	CCTGGACTGTAGCAAAGCGGT	21	
C.cajan_16563	*CcWRKY29*	F	CTTCCACCACCATGCTCCGT	20	184
		R	ACCACCCTCTTCCTCTGCCT	20	
C.cajan_25148	*CcWRKY30*	F	CCTCAAAGCATCGCCGCCA	19	179
		R	GTTGCTTTGTTGACGGCGGC	20	
C.cajan_25149	*CcWRKY31*	F	GCTTCCCTTTCTCCCCTGCTTT	22	167
		R	TGGTGGTGCTGATGTGGTGAC	21	
C.cajan_04816	*CcWRKY32*	F	GCGTCTCCTCCAACTCCTC	19	150
		R	GGCACCGCTTCTTGAACG	18	
C.cajan_41239	*CcWRKY33*	F	CTCTCGGCCTCCAACAAGTG	20	200
		R	GGACACCCTCTCACCGTACT	20	
C.cajan_05144	*CcWRKY34*	F	CCTCCTCCTGTTCCTGCTCC	20	159
		R	CGCACCCTTCCCCTTCCTTA	20	
C.cajan_05149	*CcWRKY35*	F	ACTGTCTTCAAAGGGTGGGG	20	150
		R	GTAACTCCCCTCCGTCCAAG	20	
C.cajan_04448	*CcWRKY36*	F	GCAACAGCAACGCATGGAAC	20	173
		R	TGGGAGGTGTGCTTGATCGA	20	
C.cajan_16799	*CcWRKY37*	F	GGTGTTGGTGGAGGGAAGGT	20	158
		R	ACGCCATCTCTTGTTCCACCT	21	
C.cajan_2733	*CcWRKY38*	F	CCCAAGGCATCCAAACCAGC	20	183
		R	ACCTGCTGAGAGTGGCCAC	19	

C.cajan_35299	*CcWRKY39*	F	TTCCAAATCCCCTTCCCCGG	20	187
		R	TGTCTGCAAGGTGTGGTGGT	20	
C.cajan_17992	*CcWRKY40*	F	GGCCAGAAACAGGTGAAGGG	20	181
		R	TGATGGTGTCTTTGGAGCGC	20	
C.cajan_04900	*CcWRKY41*	F	AACGTCCTCATCCCATGCCT	20	159
		R	TGGCGTCAGTGTTTCTCCTCT	21	
C.cajan_05822	*CcWRKY42*	F	CCTCGCTTTGCCTTCTTGACT	21	154
		R	CCACCCTTTTCTTCACACCACA	22	
C.cajan_42797	*CcWRKY43*	F	GCGCGCACTCCATGTATG	18	172
		R	TCCACCCGAACTCCCTGA	18	
XM_020367686.1	Actin	F	TGTTCCAGCCATCCATGA	18	180
		R	CCAACGCGGTGATTTCTT	18	
XM_020353202.1	β-Tubulin	F	GCCCTGACAACTTCGTCTTC	20	125
		R	GCAGTTTTCAGCCTCTTTGC	20	

Table S8: Comparative expression profile of individual pigeonpea WRKY genes that are either highly or lowly up-regulated under individual stresses of *Fusarium udum* and NaCl

**Highly up-regulated in *F. udum* (> 10 folds) but lowly up-regulated in NaCl (< 10 folds)**	**Highly up-regulated in NaCl (> 10 folds) but lowly up-regulated in *F. udum* (< 10 folds)**	**Highly up-regulated in both individual stresses of *F. udum* and NaCl (> 10 folds)**	**Lowly up-regulated in both individual stresses of *F. udum* and NaCl (< 10 fold)**
CcWRKY 1<br>*CcWRKY 36*	*CcWRKY 10*<br>*CcWRKY 21*<br>*CcWRKY 23*<br>*CcWRKY 28*<br>*CcWRKY 29*<br>*CcWRKY 31*<br>*CcWRKY 34*<br>*CcWRKY 37*<br>*CcWRKY 38*	*CcWRKY 4*<br>*CcWRKY 7*<br>*CcWRKY 12*<br>*CcWRKY 22*<br>*CcWRKY 30*<br>*CcWRKY 32*<br>*CcWRKY 39*<br>*CcWRKY 43*	*CcWRKY 5*<br>*CcWRKY 14*<br>*CcWRKY 24*<br>*CcWRKY 25*<br>*CcWRKY 27*<br>*CcWRKY 33*<br>*CcWRKY 42*
